# Numerical Modelling of Keratinocyte Behaviour: A Comprehensive Review of Biochemical and Mechanical Frameworks

**DOI:** 10.3390/cells14171382

**Published:** 2025-09-04

**Authors:** Sarjeel Rashid, Raman Maiti, Anish Roy

**Affiliations:** Wolfson School of Mechanical, Electrical and Manufacturing Engineering, Loughborough University, Loughborough, Leicestershire LE11 3TU, UK; r.maiti@lboro.ac.uk (R.M.); a.roy3@lboro.ac.uk (A.R.)

**Keywords:** keratinocytes, modelling, wound healing, Finite Element Analysis (FEA), mechanobiology, Machine Learning (ML)

## Abstract

Keratinocytes are the primary cells of the epidermis layer in our skin. They play a crucial role in maintaining skin health, responding to injuries, and counteracting disease progression. Understanding their behaviour is essential for advancing wound healing therapies, improving outcomes in regenerative medicine, and developing numerical models that accurately mimic skin deformation. To create physically representative models, it is essential to evaluate the nuanced ways in which keratinocytes deform, interact, and respond to mechanical and biochemical signals. This has prompted researchers to investigate various computational methods that capture these dynamics effectively. This review summarises the main mathematical and biomechanical modelling techniques (with particular focus on the literature published since 2010). It includes reaction–diffusion frameworks, finite element analysis, viscoelastic models, stochastic simulations, and agent-based approaches. We also highlight how machine learning is being integrated to accelerate model calibration, improve image-based analyses, and enhance predictive simulations. While these models have significantly improved our understanding of keratinocyte function, many approaches rely on idealised assumptions. These may be two-dimensional unicellular analysis, simplistic material properties, or uncoupled analyses between mechanical and biochemical factors. We discuss the need for multiscale, integrative modelling frameworks that bridge these computational and experimental approaches. A more holistic representation of keratinocyte behaviour could enhance the development of personalised therapies, improve disease modelling, and refine bioengineered skin substitutes for clinical applications.

## 1. Introduction

Keratinocytes are the predominant cells of the topmost layer of our skin, called the epidermis. These cells serve as the skin’s first line of defence. In addition to forming a physical barrier, they serve as active participants in tissue maintenance, immune response, and wound healing. The ability to respond dynamically to environmental cues (mechanical stress, chemical signals, pathogens, etc.) makes these cells critical to both homeostasis and disease progression.

When keratinocyte functionality is disrupted, the clinical implications are substantial. Chronic wounds, particularly those associated with diabetes, venous insufficiency, or pressure ulcers, are notoriously difficult to heal and are linked to high rates of morbidity. In the United Kingdom alone, managing these hard-to-heal wounds costs the National Health Service approximately £10 billion annually, a figure that is projected to rise with ageing populations and increased diabetes prevalence [1,2,3] Conversely, when keratinocyte dynamics are pathologically accelerated or dysregulated, the consequences can be equally severe. Uncontrolled proliferation and differentiation influence chronic inflammatory skin diseases such as psoriasis. In this condition, keratinocytes exhibit hyperresponsiveness to messenger proteins in the immune system called cytokines. They can also resist apoptosis (cell death). This leads to epidermal thickening and immune infiltration [4]. Moreover, unchecked keratinocyte expansion can drive malignant transformation into squamous cell carcinoma (SCC), one of the most common forms of skin cancer. Recent work highlights how epidermal barrier genes and metabolic regulators, such as fructose-1,6-bisphosphatase 1 (FBP1) and regenerating islet-derived protein 3 alpha (REG3A), modulate the delicate balance between keratinocyte proliferation and differentiation. These are mechanisms that, when disrupted, pave the way for both inflammatory disorders and tumorigenesis [5].

Considering this delicate balance, where either insufficient or excessive activity of keratinocyte pathways can result in disease, a detailed and mechanistic comprehension of how keratinocytes perceive microenvironmental signals (like substrate rigidity, cytokines, and electrical gradients) is crucial. Such insights hold promise not only for advancing dermatological therapies but also for designing next-generation tissue-engineered skin grafts, transdermal drug delivery platforms, and digital twins for precision wound care.

This review aims to present a panoramic survey of numerical approaches that investigate keratinocyte behaviour from molecule to macroscale.

We first start with laying the foundation of keratinocyte biology, along with providing the motivation for modelling their behaviour and existing gaps in the literature (Section 1). We then catalogue the mathematical relations: reaction–diffusion (R-D), agent-based, and continuum (Section 2). We then examine how mechanobiological inputs such as substratum stiffness, electric fields (EFs), and the adhesion between the cell and the extracellular matrix (ECM) are incorporated (Section 3). Section 4 focuses on finite element analysis (FEA) applications, highlighting single-cell atomic force microscopy (AFM) indentation to multicellular constructs. Section 5 discusses emerging trends, including machine learning (ML)-assisted model calibration, organ-on-chip validation, and patient-specific digital twins. We close by outlining future directions for coupling stochastic and continuum models with data-driven surrogates to achieve predictive fidelity, and summarising key take-home messages (Section 6).

### 1.1. Basics of Keratinocyte Biology

**Figure 1:**
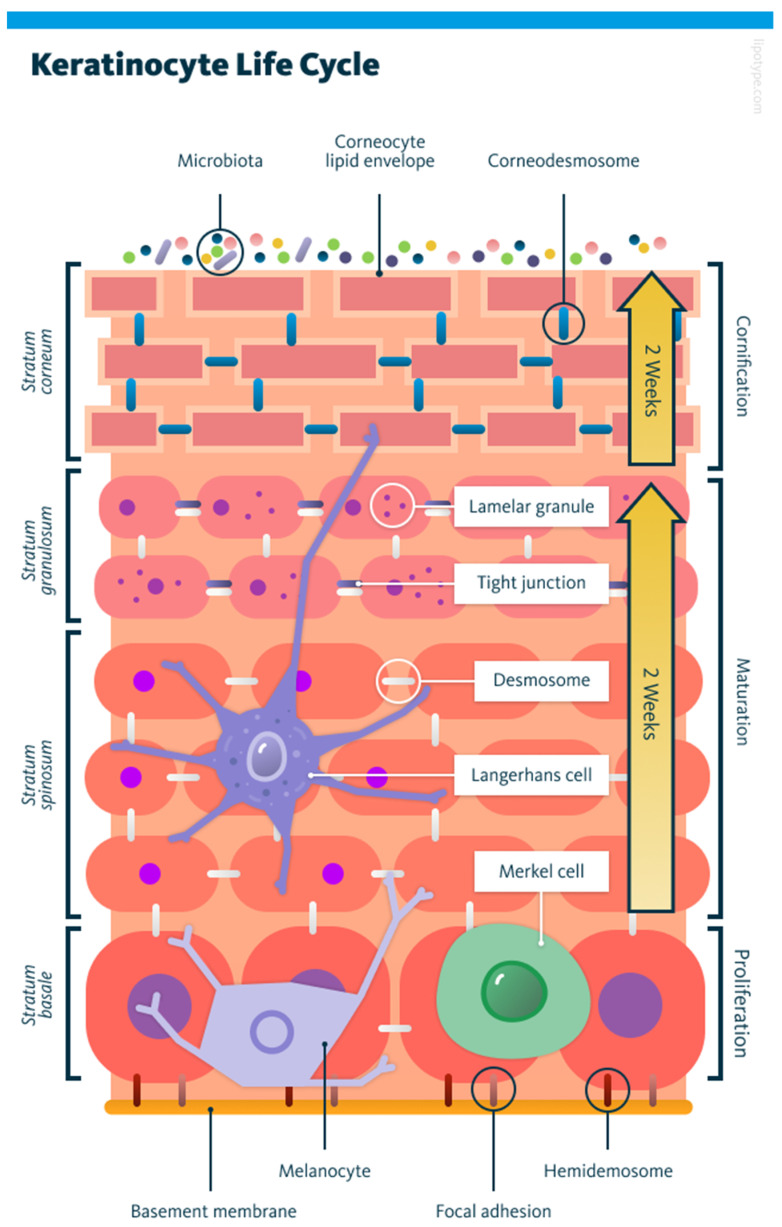
Illustration of keratinocyte progression through the epidermis, from proliferation in the basal layer to maturation and cornification in the upper layers. Background colours shift from deep pink to light orange, marking each phase. Key structures are colour-coded. Microbiota are represented by multicoloured dots. Lamellar granules are pink with purple dots. Tight junctions are blue rectangles with rounded corners. Desmosomes are shown in white. Various cell types are also included: Langerhans cells are purple, Merkel cells are green, and melanocytes are light purple. Two large yellow arrows indicate the timeline of upward migration: two weeks from the stratum basale to the granulosum, and another two weeks to reach the stratum corneum. Adapted from [7].

The human epidermis undergoes complete renewal every three to four weeks through a finely regulated, conveyor belt-like process governed by the proliferation, differentiation, and migration of keratinocytes, the predominant cell type in the skin’s outermost layer. The epidermis itself consists mostly of keratinocytes, which arise from stem cells in the basal layer (stratum basale). As the keratinocytes progress upward, these pass through intermediate layers: spinous (stratum spinosum) and granular (stratum granulosum). The whole process involves gradual differentiation. Throughout this migration, keratinocytes produce lipid-rich lamellar bodies that contribute to the skin’s protective barrier. Eventually, the keratinocytes lose the nuclei and become flattened corneocytes embedded in lipid layers, forming the robust, brick-and-mortar-like structure of the stratum corneum that acts as the body’s primary physical and immunological barrier [6]. The layered progression is illustrated in Figure 1.

This renewal process is not only vital for maintaining hydration and blocking pathogen entry, but also plays a crucial role in wound healing, protection from ultraviolet rays, and sensory function. Slowing down of this cellular process, whether due to chronic wounds, metabolic disorders, or age-related degeneration, can compromise skin integrity and lead to significant healthcare challenges. Examples include inflammation and infection [8], immune dysfunction [9], and increased risk of lower limb amputation [10], culminating in frequent hospitalisations [11,12].

**Figure 2:**
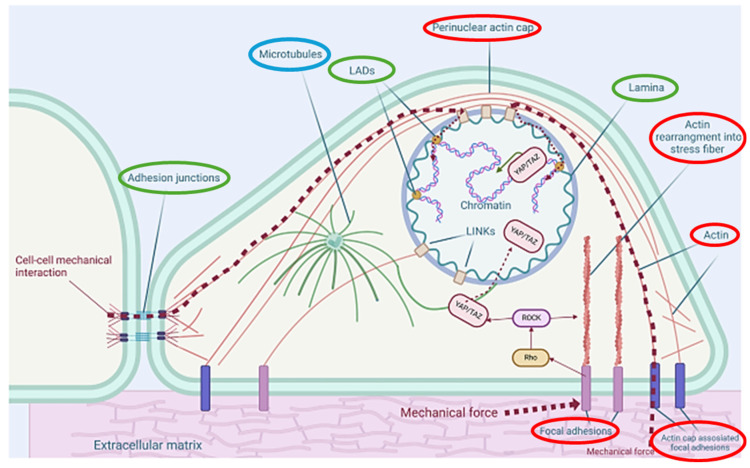
Typical cytoskeletal organisation showing actin filaments forming stress fibres, the perinuclear cap and FAs (all in red), structures made from IF’s (green), and microtubules (blue). Adapted from [13].

Beyond serving as a protective barrier, keratinocytes contain a complex internal structure crucial for their mechanical and biological functions. As shown in Figure 2 [9], the cytoskeleton consists of actin filaments approximately 6–8 nm), intermediate filaments (IF’s) (approx. 10 nm), and microtubules (approx. 25 nm) [13]. Shown in red are the actin filaments and the structures that form, such as the perinuclear actin cap and stress fibres. This enables the keratinocytes to generate contractile muscular forces (via myosin motors), maintain cell shape, and form focal adhesions (FAs) that link the cytoskeleton to the ECM [14]. Microtubules (shown in blue) extend radially from the centrosome and control intracellular transport, spatial organisation of organelles, and communication with the nuclear envelope [15]. IF’s and their related structures have been shown in green. These are the nuclear lamina and lamina-associated domains (LADs) which span the cytoplasm, anchor to desmosomes at (cell–cell) adhesion junctions, and distribute mechanical stresses across the tissue [16]. Another important IF is keratin (not explicitly shown), that reinforces cell structure and contributes to keratinocyte stability and resilience.

These networks continuously reorganise in response to external cues like substrate stiffness and mechanical forces, forming the foundation for the mechanical models explored in this review.

A schematic representation of these cytoskeletal components within a typical eukaryotic cell like a keratinocyte is shown in Figure 2, with the core structures emphasised.

### 1.2. Motivation for Modelling Keratinocyte Behaviour

In the last twenty years, in vitro assays such as scratch wound migration plates, three-dimensional organotypic cultures, and AFM micro rheology have uncovered numerous biochemical pathways that direct keratinocyte behaviour. For instance, scratch wound assays remain a widely adopted tool to quantify keratinocyte migration and re-epithelialisation dynamics [17]. Enhanced quantification and reproducibility can be achieved through modifications in furrow patterning. This involves creating precise grooves or patterns in the substrate to guide cell migration. Additionally, live-cell tracking further contributes to these improvements, as highlighted in [18]. By implementing these strategies, researchers can obtain more accurate and reliable data to understand cell behaviour.

**Figure 3:**
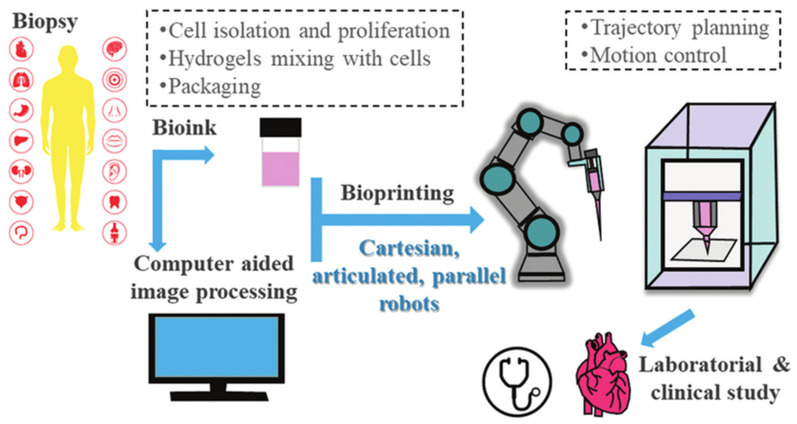
Schematic outlining the workflow from initial biopsy to clinical application. It begins with cell isolation and proliferation, followed by mixing cells with hydrogels and packaging. Computer-aided image processing then prepares the bioink which is used in bioprinting via robotic systems. The three types are Cartesian, articulated, or parallel. This stage involves trajectory planning and motion control to ensure precision. The final printed constructs are tested under lab and clinical conditions to validate functionality and safety. Adapted from [23].

Yet three persistent shortcomings remain. First, most assays are performed on flat, overly stiff plastic surfaces that fail to replicate real skin’s compliant, anisotropic architecture [19]. Secondly, high-content imaging often produces snapshot correlations rather than causal mechanisms. For instance, determining whether YAP/TAZ activation is a driver or a consequence of keratinocyte migration remains challenging without controlled perturbation strategies [20]. Third, real-life clinical scenarios often involve complex mechanical or electrical stimuli, such as the shear forces beneath wound dressings or endogenous EFs in reepithelialising wounds, that are challenging to mimic in vitro [21]. Recently, robotics-assisted bioprinting and automated live-cell imaging platforms are now emerging to tackle these challenges with higher reproducibility and throughput [22]. The basic methodology is summarised in Figure 3.

To complement these experimental studies, researchers have turned to numerical models that recapitulate keratinocyte dynamics across length and timescales. R-D frameworks reproduce calcium wave propagation and oxygen deprivation around pressure ulcers. Agent-based models (ABMs) simulate clonal expansion, i.e., the selective outgrowth of a mutant keratinocyte stem-cell clone that progressively replaces neighbouring cells. They also show the interplay between proliferation and differentiation [24] while continuum formulations capture collective cell migration as an active fluid [25]. To resolve local stress and strain during AFM indentation or microneedle penetration, FEA can be used [26]. Such in silico tools can test in silico mutations, apply virtual forces or EFs, and run dozens of parameters sweeps for long durations on tasks that are impractical in a clinical setting.

Despite this progress, the field is fragmented. Many chemical models neglect mechanics, while mechanical models often assume homogenous cell populations. There are very few frameworks that bridge single-cell phenomena to tissue-scale outcomes, leading to lingering controversies regarding the approach to modelling. For example, some continuum models predict that low substratum stiffness slows keratinocyte migration because of weaker traction force. However, AFM-calibrated FEA and in vivo studies have shown that soft hydrogels can boost migration through PIEZO1, a mechanosensitive ion channel protein that influences retraction dynamics and cytoskeletal activity [27]. This results in accelerated wound closure, contradicting earlier models [28,29]. In electrotaxis (directed cell movement in response to an external electric field), there is still debate on whether polarity bias or speed bias influences keratinocyte movement in EFs created by wounds, with experimental differences and complex ion–cytoskeleton interactions leading to varied interpretations [30].

Moreover, many existing computational and experimental studies often address only isolated aspects of keratinocyte behaviour. For instance, their response to either biochemical cues or mechanical forces in simplified 2D environments. In reality, keratinocytes function within a dynamic 3D microenvironment where substrate stiffness, chemical gradients, and EFs all contribute to influencing migration, proliferation, and even cancerous metastasis. This incomplete perspective restricts the ability to predict keratinocyte responses in chronic wound healing, transdermal drug delivery, and other such applications. There is a need for more integrative models that capture both mechanical and biological processes in a more realistic multicellular arrangement. Addressing issues like this is vital for improving existing therapies with harmful drawbacks. For instance, while targeted blue light emitting diode (LED) therapy helps combat bacterial infections, it also triggers transient morphological changes in keratinocytes [31]

By mapping the current landscape and highlighting disparities, we aim to guide both modellers and experimentalists toward integrated platforms that can accelerate wound healing therapies, refine toxicity testing, and aid in understanding the pathophysiology of skin disease.

## 2. An Overview of Modelling Techniques

Keratinocytes operate within a complex environment of biochemical signals, mechanical forces, and intercellular interactions. To understand how these cells orchestrate wound healing, barrier function, and disease processes, researchers have increasingly turned to computational models. Such models not only capture the intricate spatial and temporal dynamics of molecular signalling but also enable in silico experiments that would be difficult, costly, or impossible to conduct in vivo [32]. Before exploring the mechanical behaviour of keratinocytes in detail, it is essential to establish the mathematical and computational frameworks that form the backbone of modern cellular modelling. This section, therefore, reviews the fundamental approaches. Namely, R-D equations, stochastic and kinetic simulations, ABMs, and continuum multiphase methods. These are the starting points to simulate keratinocyte behaviour. These diverse techniques set the stage for later sections, where we examine how realistic mechanical cues, finite element (FE) modelling, and emerging computational tools can be integrated to reveal the physical principles governing keratinocyte function.

### 2.1. Reaction Diffusion Frameworks

R-D models describe how concentrations of one or more biochemical species change in space and time under the combined influence of molecular reactions and Fickian diffusion. It is a mass transport process characterised by the movement of particles from areas of high concentration to low concentration, leading to a uniform distribution over time. Mathematically, these expressions take the following form:

(1) $$\frac{\partial C_{i}}{\partial t}=D_{i}\nabla^{2}C_{i}+R_{i}(C_{1},\ldots ,C_{n},x,t)$$

where $C_{i}$ denotes the concentration of species i, $D_{i}$ its diffusion coefficient and $R_{i}$ is a nonlinear reaction term that has a spatial coordinate x, and time t.

The epidermis is a shallow and densely packed epithelium. It often exhibits keratinocyte-centred phenomena that occur on length scales of 10–500 µm and timescales ranging from seconds to hours. These processes are well captured by continuum partial differential equations.

The first keratinocyte-centred models focused on pressure-driven hypoxia. Sree et al. [33] coupled Equation (1) (for oxygen) to a ‘switch’ that flips keratinocytes from proliferation to apoptosis once pressure of oxygen drops below a threshold. This phenomenon is common in pressure ulcers. Numerically, the authors used an implicit finite difference scheme in MATLAB, allowing millimetre-scale domains to run in seconds. The strength in the model lies in the fact that one species and one threshold can reproduce biopsy-verified necrotic rings. However, the model assumes a direct, linear relationship between external pressure and oxygen decrease, overlooks any spatial mechanics, and exhibits high sensitivity to the “keratinocyte chemoattractant” parameter. The authors, therefore, called for fully three-dimensional models that have more mechanical realism.

Significant research has also been conducted in terms of wound healing. Near-instantaneous injury signals require extremely accurate temporal resolution. Odagiri et al. [34] built a hybrid model. This involved the following:

1. Cellular Potts Model: a technique where cell behaviour is simulated using a lattice structure.
2. Partial Differential Equations (PDEs): used to define concentrations of various chemicals using continuous fields.

In this framework, each lattice node secretes the energy carrier molecule called adenosine triphosphate (ATP) when “wounded”. The ATP diffusion (modelled by explicit forward Euler), triggers Calcium ions (Ca^2+^) to be released via a PDE membrane model. This subsequently feeds an energy function within the Cellular Potts Model that moves the cells. Despite the manual method of parameter fitting, wave speed and amplitude results are shown to match the experimental micro-particle image velocimetry (micro-PIV) recordings. Odagiri et al. [35] have scaled the same framework to a centimetre-scale slab and incorporated a finite-volume Graphics Processing Unit (GPU) solver. They observed that increasing ectonucleotides activity, which are enzymes that generate substances relevant to immune and inflammatory responses, leads to a reduction in ATP/Ca^2+^ wave radii. Additionally, this increased activity accelerates closure by suppressing edge instabilities. Both variants, however, consider diffusion to be isotropic and spatially uniform. Furthermore, the eventual maturation of tight junctions means that substances will be more effectively blocked from moving between cells [36]. This reduction in cell–cell permeability has not been accounted for, so this assumption is likely to overestimate how far calcium signalling can influence cells across the epidermal layers.

**Figure 4:**
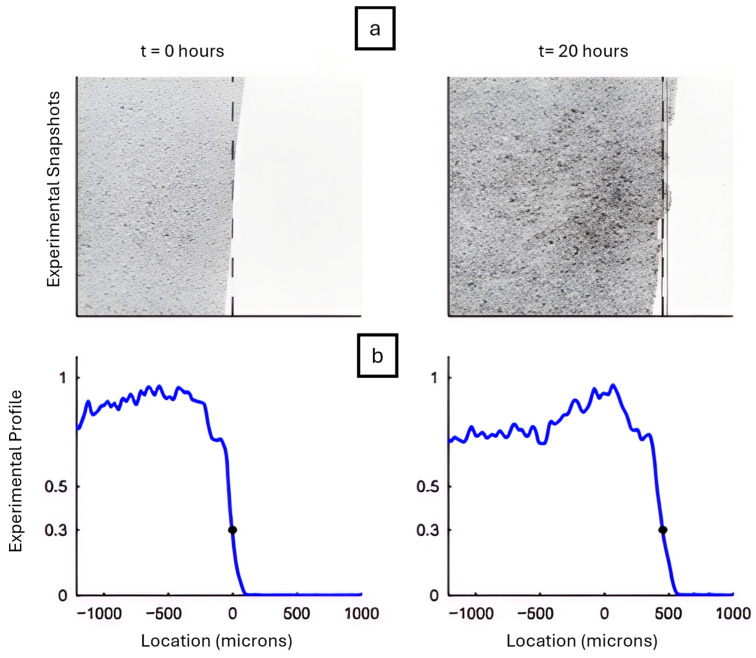
Top row (**a**) shows snapshots at t = 0 and t = 20 h, while the bottom row (**b**) shows the corresponding experimental density profiles. Dashed lines in (**a**) indicate the calculated leading edge position by the model. Dots on the density profiles in (**b**) denote the computed values of the cell density at that leading edge location from the reference (zero) position, measured in microns. The y axis in (**b**) represents the normalised cell density in the keratinocyte monolayer. Adapted from [37].

Chemical fields are not the only drivers of cell motion. Nardini et al. [37] retained the PDE structure of Equation (2) but set the diffusion coefficient, D as

(2) $$\;D\left(u\right)=\alpha u\left(1-u\right)$$

where u is local cell density. A positive α mimics adhesion-induced “drag”, while a negative value of α mimics adhesion-assisted “pulling”. This model used a Crank–Nicolson solver, which is a numerical method that accurately solves time-dependent diffusion equations by averaging values between time. It revealed that a time-varying parameter, α(t) is required to fit experimental scratch-assay fronts. This framework was used to reproduce keratinocyte migration in scratch wound assays, where a cell sheet advances into an initially cell-free gap. As shown in Figure 4, the leading edge moves significantly between t = 0 and t = 20 h, a process captured by adjusting α(t) over time.

The equation used connects classical R-D and continuum mechanics and allows for linear stability analysis. It highlights the need to account for evolving adhesion dynamics in continuum models. However, it condenses all adhesion physics into a single parameter. This approach overlooks the turnover of E-cadherin, a protein that maintains the structure and integrity of epithelial tissues, as well as substrate effects.

To address additional biochemical influences on keratinocyte behaviour, subsequent models have integrated growth factor signalling into the R-D framework. Andasari et al. [38] applied a semi-implicit finite difference solver. They showed that fibroblast-secreted epidermal growth factor (EGF) delays keratinocyte advancement until integrin expression recovers, which involves proteins connecting the cell cytoskeleton to ECM. A sensitivity test using a method called Latin Hypercube sampling was used. The sampling method ensured the correct determination of Keratinocytes in the multidimensional space. It showed that the EGF degradation rate and integrin binding affinity are the most influential factors on skin re-epithelialisation.

**Figure 5:**
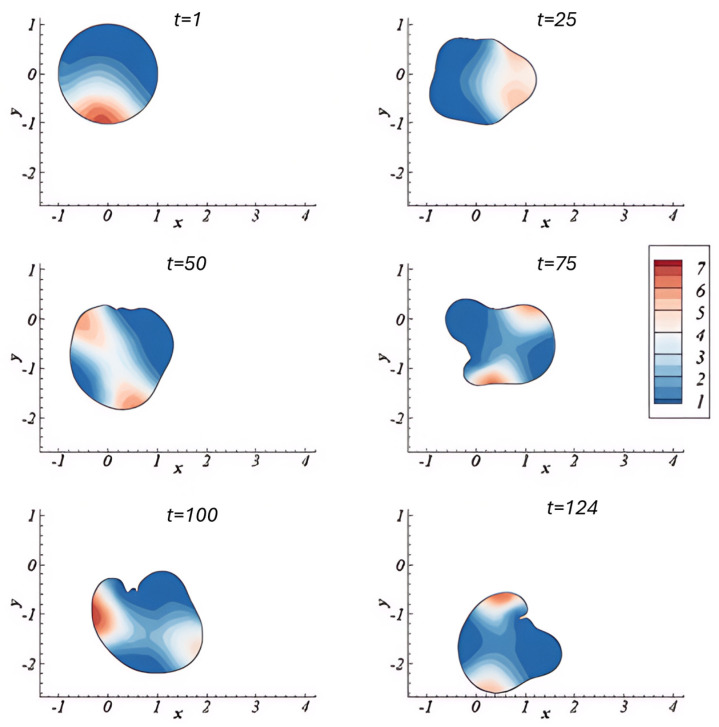
Spontaneous cell migration driven by internal actin dynamics shown at time intervals in seconds, starting from t = 1 to t = 124. Localised regions of high $\alpha_{b}$ (red) correspond to actin-rich protrusive zones, while low $\alpha_{b}$ (blue) correspond to retractive regions. The x and y axes represent spatial coordinates. Adapted from [39].

Madzvamuse et al. [39] incorporated mechanical properties by formulating a bulk–surface framework to simulate 3D cell migration. This framework models and couples the interaction occurring at the cell or tissue surface boundary. This was conducted by integrating biochemical signalling with mechanical deformation. The model treats the cell membrane and cytoskeleton as dynamically coupled domains and uses a moving mesh to capture membrane motion and a viscoelastic, contractile model for cytosolic mechanics. R-D systems were implemented to represent actin and myosin dynamics, which generate force and drive cell movement. Their framework enabled the simulation of realistic behaviours such as cell protrusion, retraction, and polarisation, and it was validated through consistency with earlier theoretical predictions [40]. Moreover, it can simulate spontaneous, self-organised migration. As shown in Figure 5, the cell begins in a symmetric, rounded state but gradually develops protrusions and directional bias over time due to polarisation of the cytosolic field, $\alpha_{b}$, which represents actin activity. The x and y coordinate axes are spatial coordinates and define the direction and movement of the cell within the physical environment at certain time intervals. These simulations demonstrate how internal instability and pattern formation can initiate directed migration, membrane deformation, and persistent motion even in the absence of spatial cues.

Although promising, the model in [39] focused on early-stage deformations and did not incorporate real-time experimental datasets or keratinocyte-specific properties, highlighting key avenues for future refinement.

At tissue scale, Asakura et al. [41] nested single-cell polarity kinetics in an active-gel Brinkman continuum. Using a coupled finite-element/finite-volume solver, they captured leader-cell emergence and stress fibre alignment under realistic physical constraints. Leader cells are those at the front that guide collective movement, while stress fibre alignment refers to the organisation of contractile actin bundles within cells in a particular direction. On the other hand, Zhao et al. [42] employed an R-D/PDE system combined with terms describing active contractile forces, such as those generated by cells to recreate actomyosin rings (circular bundles of actin filaments and myosin motors) observed in embryonic wounds. Finally, Cao et al. [43] presents a novel two-component biochemical R-D model which showed that migration modes are dependent on substrate modulus using a similar framework All three studies emphasise that coupling mechanics to chemistry changes not only numbers but qualitative cell behaviour (e.g., leader-cell fraction or traction force generation) at all scales.

While these continuum-scale models capture emergent tissue behaviours well, they often assume uniformity in cellular response behaviour. Yet biological systems, particularly keratinocyte populations, naturally exhibit significant variability in molecular turnover, mechanical feedback, and signal reception [44]. This has motivated substantial research into stochastic modelling approaches to better reflect their noisy, heterogeneous reality.

### 2.2. Stochastic and Kinetic Modelling Approaches

Keratinocyte state transitions and signalling bursts are inherently stochastic processes, driven by low molecule counts and discrete molecular events. To capture this variability, three principal modelling strategies dominate the literature: ordinary differential equations (ODEs), kinetic Monte Carlo (KMC) methods, and Langevin noise models. Each formalism strikes a different balance between biological realism and computational cost, and each lends itself to specific types of cellular questions. This section reviews their theoretical foundations and practical relevance to keratinocyte biology.

ODEs have long served as a robust technique for modelling signalling cascades. These are a series of biochemical reactions inside a cell, triggered by a signal, that lead to a specific cellular response. ODE’S can offer deterministic, continuous frameworks to track average molecular concentrations. Their efficiency and scalability make them well-suited for large systems, yet they struggle to reproduce fluctuations critical to cellular decision-making [45,46]. To extend their utility, stochastic frameworks, particularly those based on random processes, have been used to simulate keratinocyte migration behaviours. These approaches capture the variability and complexity of individual cell movements, providing insight into processes such as wound healing and cancer metastasis [47].

KMC methods simulate individual reaction events by sampling from the chemical master equation. It is a foundational mathematical model for stochastic chemical kinetics in biological systems. It captures the discrete and random nature of molecular interactions, especially at low copy numbers. This bottom-up approach accurately captures highly probabilistic switching events, such as the activation of epidermal growth factor receptor (EGFR) [48,49].

In the context of keratinocytes, Gouveia et al. [50] applied KMC to predict the distribution of keratin aggregates in mutant cells. The study showed how shape changes and reaction noise can lead to a buildup of keratin near the nucleus, a phenomenon known as perinuclear clustering. This clustering shows how the structure of the cytoskeleton and local reaction speeds influence cell adherence near the nucleus. Stochastic fluctuations help drive this process. By resolving such spatial and stochastic details, KMC offers a powerful tool for interrogating subcellular organisation. However, its computational demands limit its use in large or highly connected systems.

Langevin models bridge the gap between these extremes by incorporating noise terms into ODEs. Originally developed for ion channel gating and excitable membranes [51]. Langevin equations retain computational efficiency while approximating intrinsic and extrinsic fluctuations. While less precise than KMC, these models are useful to strike a balance for systems with moderate molecular complexity.

These main methods are summarised in Table 1, alongside several cross-categoric stochastic frameworks, which are being increasingly applied to cell migration and signalling applications. These are shown subsequently in Table 2.

Despite substantial progress within this field, several challenges remain. As previously mentioned, KMC methods are computationally expensive, particularly in systems with a high number of reactions [48]. Accurate parameterisation of stochastic models is another hurdle, due to biological variability and limited experimental data [58]. Spatial complexity compounds the issue, as incorporating it into stochastic frameworks requires additional computational effort; though hybrid methods used in other applications show promise [54].

There is a clear need for the development of hybrid models that combine deterministic and stochastic approaches, enabling better trade-offs between accuracy and performance. Crucially, experimental validation must keep pace. New tools in single-cell analysis, along with frameworks that simulate cells as discrete entities, offer key opportunities to validate stochastic predictions and improve model reliability [59].

### 2.3. Agent-Based Modelling (ABM)

Where stochastic formulations address randomness in intracellular signalling, ABMs shift focus on spatial coordination and individual cell behaviour. They offer a natural framework for simulating keratinocyte migration, clonal competition, and collective decision-making during wound healing and disease. ABMs explicitly encode cell-level rules for processes such as proliferation, adhesion, and polarity, allowing researchers to test how these mechanisms interact to shape large-scale epidermal patterns.

**Figure 6:**
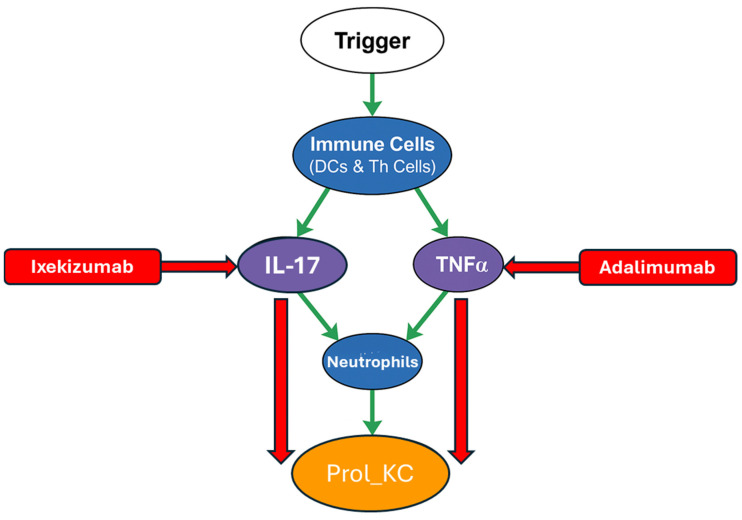
Simplified interaction map of PsoriaSys model highlighting key inflammatory feedbacks in psoriasis. Green arrows represent activation; red lines represent inhibition.

In psoriatic epidermis, ABMs have successfully reproduced hallmark features such as rete ridge elongation and hyperproliferation by simulating cytokine-driven division events and tissue thickening. These models were calibrated against in vivo data regarding turnover rates and spatial structure [60]. A compelling example of this is shown in Figure 6, which presents the model called PsoriaSys [61]. It is a multiscale Boolean population model with functional similarities to agent-based frameworks. Logical rules (AND, OR, NOT) define how node states change. It maps interactions between 87 nodes. Some of the key representations are listed below and illustrated in Figure 6:

1. Trigger: Represents the initial external or internal events that kick off psoriasis (e.g., skin injury, infections, stress).
2. Immune cell types are shown in a blue circle. For example, neutrophils, Dendritic Cells and T-helper subtypes (Th).
3. Keratinocyte state shown in an orange circle (e.g., proliferating keratinocytes (Prol_KC)). This is linked to uncontrolled growth and a key measure of disease activity in the model.
4. Major cytokines shown in a purple circle (e.g., Interleukin 17 (IL-17), Tumour Necrosis Factor Alpha (TNFα). These are inflammatory responses and are the key drivers of psoriasis.
5. Therapeutic and Regulatory Drugs/Agents shown in a red rectangle (e.g., Ixekizumab, Adalimumab).

Green arrows show activating interactions while red arrows indicate inhibition. The model illustrates how local events such as IL-17 signalling initiate keratinocyte proliferation, which leads to higher keratinocyte proliferation and tissue-level inflammation (psoriasis). As shown in silico, inhibiting IL-17 leads to a 64% reduction in Prol_KC, demonstrating the model’s predictive alignment with the clinical efficacy of IL-17-targeting biologics like Ixekizumab. Likewise, TNFα inhibition yields a 15% reduction in Prol_KC, consistent with observed therapeutic responses using Adalimumab. Please note, the complete expanded schematic of the Boolean framework is available in Appendix A.

During wound healing, proliferation dynamics are equally important, with Arciero and Swigon [62] incorporating wound geometry and cell–cell interactions to predict re-epithelialisation times across a range of wound sizes. Across both contexts, proliferation rules grounded in biological stimuli have emerged as key levers for matching model output to clinical observations.

Adhesion mechanisms influence both the structure and migration patterns of keratinocyte colonies. Simulations incorporating cell–cell and cell–substrate adhesion have shown that self-organisation and wound closure trajectories are highly sensitive to adhesion strength parameters [63]. The role of the ECM further complicates this picture. It has been demonstrated that ECM fibre alignment can promote converging migration fronts, revealing a link between matrix topology and the efficiency of collective movement [64]. Notably, both studies underline the importance of integrating microenvironmental cues into adhesion rule sets.

Cell polarity, a third major axis of ABM rule design, governs the directionality of migration. It has been shown that keratinocytes lacking specific growth factors exhibit defective polarisation and impaired motility [65]. This is a result that ABMs can readily incorporate via orientational coupling between a cell’s internal polarity vector and its migration direction. When connecting polarity dynamics to ECM feedback, it has been demonstrated that proper alignment facilitates efficient closure, creating an intersection point between adhesion and polarity rule domains [64].

Beyond biochemical and ECM cues, bioelectric fields also influence keratinocyte migration. Zhang et al. [66] combined experiments and ABMs to investigate collective electrotaxis in human keratinocyte sheets. They observed wave-like propagation of directional migration signals from the wound edge under EFs of 50–200 mV/mm. To replicate these dynamics, they developed a particle-based compass (PBC) model. It is a computational framework that treats cells as individual particles containing an internal compass that dictates their direction of movement based on external cues. Specifically, individual cells adjust their migration direction by integrating electric field vectors, free-edge guidance (migration tendency along an open edge), and mechanical interactions with their neighbours. This agent-based framework successfully reproduced experimental findings, including the speed of directional wave propagation and delays in response when the electric field polarity was reversed. Their results demonstrate how EFs can act as an additional layer of directional bias, interacting with polarity and mechanical rules to shape collective keratinocyte behaviour.

In addition to rule design, calibration strategies play a crucial role in the effectiveness of ABMs in predicting outcomes at the tissue level. Paramalingam et al. [60] validated psoriasis models against known in vivo metrics like population ratios and layer turnover, using parameter sensitivity analysis to identify key regulatory nodes. The most recent models have integrated agent-based simulations with continuum representations of cytokine diffusion or cell density, forming multiscale frameworks that improve drug target predictions [61]. Boolean abstraction is a method that simplifies biological networks by representing components as ON/OFF states and linking them through logical rules. It has emerged as an effective way to encode regulatory logic while ensuring efficient runtime in systems with high rule complexity [67].

Computational efficiency remains a practical constraint. Feess et al. [68] addressed this issue by developing ABMs with biophysically realistic inputs, such as cell density and growth rates. Their calibrated models successfully simulate wound repair dynamics without oversimplifying the core mechanics. While powerful, ABMs are not without caveats. Their inherent complexity may mask key biological drivers, and rule overspecification risks overfitting to particular datasets. Moreover, the high degree of patient-specific variability in conditions like psoriasis signals a broader need for personalised treatment approaches.

### 2.4. Continuum and Multiphase Modelling

ABMs are effective at describing discrete cell behaviours, whereas tissue-scale dynamics are much better suited by continuum and multiphase frameworks using continuous fields. These fields include cell volume fraction, velocity, or phase variables. These models display explicit cell representation, enabling the simulation of large populations by tracking spatial and temporal changes in tissue properties. The governing equation for such systems can be generalised as shown in Equation (3) [69].

(3) $$\frac{\partial \emptyset }{\partial t}=\nabla \cdot (M\nabla \mu )+S$$

where ∅ represents a phase or volume fraction, M the mobility, μ the chemical potential, and S a source term reflecting cell proliferation or loss.

**Figure 7:**
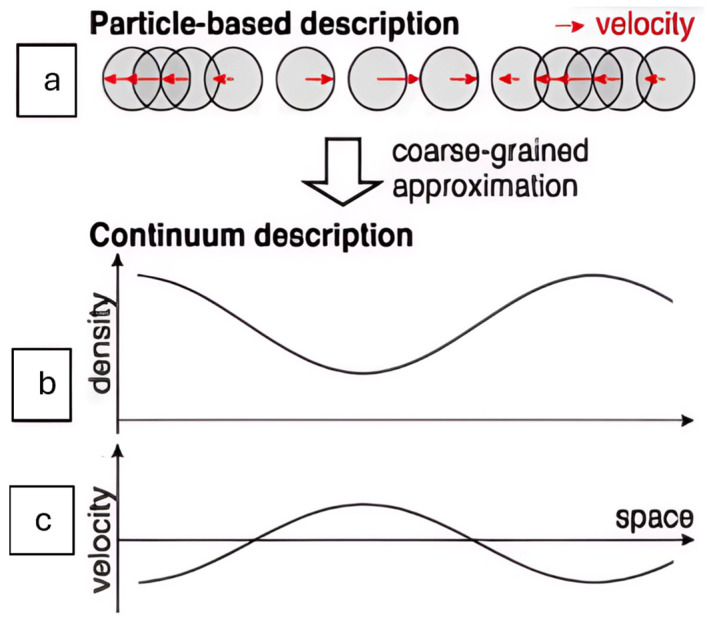
Schematic showing individual particle-based descriptions (**a**) with their velocities labelled with red arrows. They can be coarse-grained into continuous fields of density (**b**) and velocity (**c**). Adapted from [41].

To illustrate this conceptual shift, Figure 7 from Asakura et al. [41] shows how individual cell movements (discrete particles) can be approximated as continuous fields through a process called coarse graining. Figure 7a depicts individual particles and their velocities, while Figure 7b shows the corresponding continuous fields of density and velocity. This transformation provides an intuitive framework for treating tissues as deformable media rather than discrete entities.

Wenzel et al. [25] builds on this continuous field framework by using a technique called Line Integral Convolution (LIC). This method visualises vector fields like fluid velocity at high spatial resolutions. In this study, confluent cell monolayers and epithelial tissue-scale velocity fields have been visualised for four continuum-based models:

1. Random: Uncoordinated movement.
2. Elongation: Cells stretch in a particular direction.
3. Polar: Cells having a defined front-to-back direction.
4. Nematic: Cells aligning without a specific directional bias (like liquid crystals).

**Figure 8:**
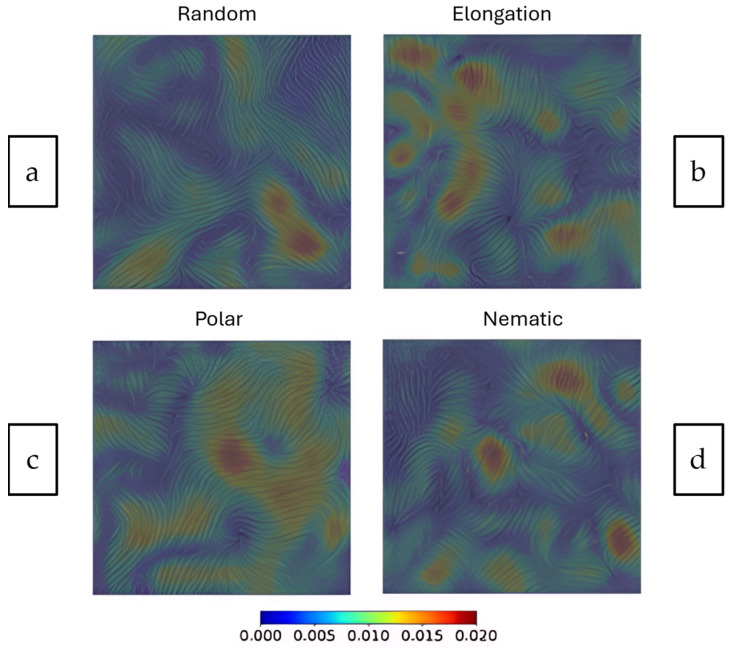
(**a**–**d**): LIC visualisation of cell dynamics under four different modelling assumptions: random (**a**), elongation (**b**)**,** polar (**c**), and nematic (**d**). Colour indicates velocity magnitude. Blue: low velocity; red: high velocity. Adapted from [25].

These swirling patterns reflect collective dynamics in confluent monolayers, where velocity magnitudes vary across space. Figure 8 demonstrates how continuum frameworks visualise large-scale behaviours like collective migration and flow coordination, offering insights that are difficult to extract from discrete representations alone.

Continuum and multiphase-field formulations have significantly advanced the understanding and prediction of collective keratinocyte behaviour, capturing migration speed, sheet buckling, and wound edge stress transmission. These models offer a more holistic framework than discrete or R-D approaches. They can simulate mechanochemical interactions and cell–cell dynamics across multiple scales. Recent studies have extended the simplified representation shown in the equation above into fully three-dimensional contexts, enabling better treatment of shape variability and force–velocity relationships within the actin network [70]. Shao et al. have incorporated membrane bending forces, surface tension, and actin bundle dynamics to simulate the changes in cell shape/size over time, i.e morphodynamics in fish keratinocytes [71].

**Figure 9:**
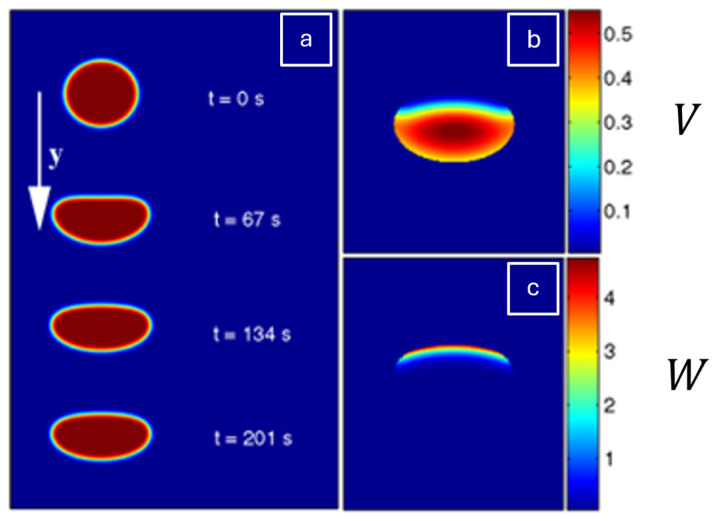
(**a**) Snapshots of the numerical evolution of a cell’s shape along spatial coordinate, y with time, t using a phase-field method. (**b**,**c**) show the steady-state distributions of auxiliary fields V (actin filaments) and W (actin bundles), respectively, which define the cell’s interior and dynamics. High V is shown in the leading edge in (**b**), while high W is shown in the trailing edge in (**c**). Adapted from [71].

A practical visualisation of their formulation is provided in Figure 9a–c, which demonstrates how cell morphodynamics can be simulated using a phase-field approach. Figure 9a shows how the cell boundary (spatial coordinate y=0 represents the cell’s centre) evolves naturally over time (t) eliminating the need for explicit interface tracking. Figure 9b,c display the steady-state distributions of auxiliary fields V (cross-linked actin filaments) and W(actin bundles), respectively. A perturbation to start the cell’s movement is performed by prescribing asymmetry in the field W (e.g., W =${\;W}_{0}y$ for y<0) causing retraction at the rear and protrusion at the front. Over time, W accumulates at the back, while a high concentration of V forms at the leading edge. This spatial organisation of actin structures drives steady motion and defines the cell’s shape and migration speed. Together, these fields illustrate how continuum models integrate intracellular dynamics to simulate keratinocyte behaviour.

These frameworks are unique in that they offer a macroscopic lens on cell migration by integrating mechanical and chemical signals. For instance, links between extracellular signal-regulated kinase (ERK) activity gradients and cell density have been central to explaining coordinated migration in wound repair [25]. Multiphasic-field models can also simulate transitions between jammed and fluid-like states in epithelial layers by capturing cell deformations and tissue-level flows. For example, three-dimensional phase-field simulations based on Ginzburg–Landau theory and spherical harmonics have been used to model motility of spherical cells and emphasise dynamic shifts in large-radius populations [72]. These advanced mathematical frameworks describe complex physical phenomena, such as phase transitions or shapes of objects in 3D space, respectively.

Additionally, continuum frameworks have begun to incorporate bulk–surface coupling principles and dynamic boundary conditions, though many remain limited in their ability to represent interface mechanics or cytoskeletal feedback [73]. Quantitative comparisons with live imaging and traction force microscopy (TFM) have reinforced model credibility. Notably, both Zhang et al. [66] and Wenzel et al. [25] successfully reproduced migration trajectories and stress transmission patterns in keratinocyte sheets despite differences in their numerical implementation.

Even minor perturbations in input values can propagate to wide variations in model output. Brückner et al. [74] emphasise the sensitivity of continuum simulations to parameter noise, raising concerns over robustness. Additionally, continuum models often assume regional uniformity, neglecting important intra-population variance. Janshoff et al. [75] notes that the interplay between cell-generated forces and microenvironmental response is under-represented. Furthermore, these models are also known to miss details for intra-population variance. Building on single-cell RNA sequencing (RNA-seq) has revealed four keratinocyte clusters that diverge in abundance and function across wound types [76], highlighting a gap in most existing field-scale models. This need for models that can account for individual cell behaviours within a collective context in wound healing has also been demonstrated by Liu et al. [77].

While continuum and multiphase-field models scale effectively to tissue-level phenomena, they often lack the resolution required to capture cell identity and stochastic heterogeneity. In this regard, discrete and R-D frameworks retain a distinct advantage. Yet, to understand how keratinocytes interpret and respond to external physical cues, a more focused perspective is needed. This includes examining how they spread in response to substrate stiffness, migrate under EFs, and transmit forces through the ECM. The following section critically examines how various modelling strategies have approached these mechanobiological processes, and where they continue to fall short.

## 3. Mechanobiology of Keratinocytes

Keratinocytes sense mechanical stimuli through a variety of mechanosensitive molecules, including integrins, stretch-activated ion channels like PIEZO1 protein, G protein-coupled receptors, and growth factor receptors [78]. These proteins serve as primary sensors, converting environmental changes into intracellular signals. A key structure in this process is the FA, a protein complex that links ECM to the cytoskeleton and facilitates the transmission of mechanical cues into the cell [78].

The mechanical stimulus is further relayed through the ECM–integrin–cytoskeleton–nucleus axis. Proteins such as Talin, Vinculin, and Paxillin help transmit forces, while others like VASP, Zyxin, and actinins regulate actin organisation and movement. These pathways extend to the nucleus, where mechanical signals influence gene expression and cell fate [79].

Downstream, signalling cascades such as mitogen-activated protein kinase (MAPK) pathways play an essential role in translating mechanical input into biological outcomes like migration, proliferation, and differentiation [80]. This integrated signalling network enables keratinocytes to adapt to mechanical changes in their environment, playing a crucial role in skin homeostasis and wound healing.

Computational and mathematical models are indispensable tools for dissecting the complex interplay between mechanical signals and human keratinocyte behaviours. These models offer diverse frameworks to simulate cellular responses across different scales, providing valuable predictions and highlighting areas of disagreement. This section delves into specific modelling techniques used to translate mechanical cues (stiffness, EFs, and intercellular forces) into keratinocyte behaviours.

### 3.1. Substrate Stiffness

Substrate stiffness plays a pivotal role in regulating keratinocyte behaviours, influencing migration, proliferation, differentiation, and collective coordination through a mechanotransductive network that spans the ECM integrins, cytoskeleton, and nucleus [81]. Keratinocytes detect and respond to mechanical cues via FAs, which transmit forces that ultimately influence nuclear architecture and gene expression. This includes structural protein changes such as lamin A/C levels, which are nuclear envelope proteins that regulate nuclear stiffness and gene activity [81]. Similar cytoskeletal and nuclear deformation responses have been documented in endothelial cells exposed to frictional and shear forces from cardiovascular devices, highlighting universal principles of epithelial mechanotransduction under mechanical stress [82].

Experimental studies consistently show that mechanical context shapes cellular behaviour: for example, keratinocytes cultured on soft polyacrylamide (PA) gels (1.2 kPa) migrate significantly faster (1.13 ± 0.25 µm/min) than those on stiffer gels (24 kPa), where speed decreases to 0.86 ± 0.27 µm/min [83]. While some models predict that increased stiffness enhances migration [84,85], the data also highlights the complexity and sometimes counterintuitive nature of stiffness–migration relationships. For example, although the mechanosensing model effectively replicates symmetry breaking (the start of front–back polarity for directional movement) on elastic substrates [86], it struggles to account for the nonlinear behaviours observed in softer environments.

**Figure 10:**
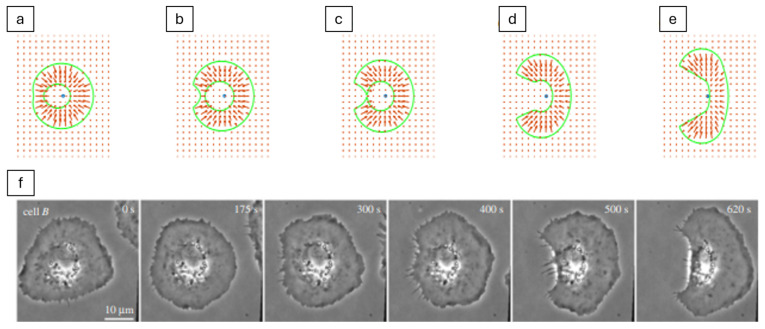
Symmetry breaking and motility onset in a keratocyte. (**a**–**e**) Modelled shape evolution driven by substrate stress; (**f**) experimental images showing matching morphological transitions. Time period from 0 s to 620 s. Adapted from [86].

This process of symmetry breaking is illustrated in Figure 10, which visualises how a keratocyte transitions from a non-motile, symmetric shape to a crescent-like migrating form. The model operates entirely through active mechanosensing. The cell probes its environment and responds to local substrate stress. Figure 10a–e show how protrusion is favoured in regions of tension and retraction in regions of compression, resulting in front–back polarity and persistent motion. The shape evolution emerges from an initial asymmetry and reinforces itself through mechanical feedback. Figure 10f presents time-lapse experimental images that closely match the model’s predictions, highlighting the accuracy and relevance of this mechanosensitive framework.

More recent frameworks have attempted to capture oscillatory motion at the single-cell level. An example of this is the self-propelled deformable particle model by Nwogbaga et al. [87], which simulates cells as individual particles that can move on their own and change shape. However, collective behaviours and context-dependent traction responses remain difficult to simulate accurately.

Leader-cell emergence and turnover are key features of directed collective migration. These are also modulated by mechanical inputs, though most models focus on biochemical signalling. Experimental evidence shows that activation of proteins like p53 and p21 plays a crucial role in initiating and resolving leader-cell phenotypes [88]. Yet, substrate mechanics likely modulate this process indirectly. Studies on epithelial monolayers suggest that more elastic substrates enhance monolayer fluidity and support leader-cell formation by reducing resistance to traction propagation [89]. This has been shown through a stochastic lattice spring model [89], which simulates cell spreading as a function of substrate stiffness and ligand density. Simulations reveal that at low stiffness and high ligand density, viscoelastic substrates promote greater cell spreading compared to purely elastic ones, challenging the traditional view that cells respond solely to instantaneous substrate stiffness. Specifically, this supports in vitro observations whereby keratinocytes on soft bases exhibit not only faster migration but also greater collective coordination. These cells form more numerous and densely populated colonies, with smaller spread areas and pronounced local deformations that facilitate cell recruitment [83]. Such cooperative behaviours are supported by increased beta-4 (β4) integrin expression at colony edges but are largely absent in keratinocytes on stiff substrates.

Beyond migration, stiffness influences proliferation and differentiation in a more nuanced, context-dependent manner. Proliferation appears enhanced on stiffer substrates, such as polydimethylsiloxane (PDMS), potentially via ERK1/2 signalling pathways [81]. In contrast, softer environments favour faster colony formation and enhanced cellular cooperation, supporting tissue spreading rather than expansion [83]. These findings reveal that keratinocytes do not respond to stiffness in a uniform manner but instead activate distinct programmes depending on the task at hand. Soft substrates appear optimised for processes like wound re-epithelialisation, while stiffer substrates favour cell cycle progression and mass regeneration. Yet, most computational models treat proliferation as a passive, density-driven process, rarely distinguishing it mechanistically from migration [38]. Keratin dynamics, too, are only partially captured. While models explore actin–keratin coupling and filament turnover [50,84] the effects of matrix stiffness on keratin organisation, especially in mutant or differentiating cells continues to remain underexplored.

Despite progress, notable gaps persist between simulations and biological observations. Many models rely on simplified assumptions, omit viscoelastic substrate effects, or are limited to specific cell lines such as human adult low calcium temperature (HaCaT) keratinocytes. Experimental variability further complicates interpretation: differences in cell source, substrate composition (e.g., PA vs. PDMS vs. glass), and two-dimensional (2D) versus three-dimensional (3D) culture conditions all affect results and limit comparability [90]. Nonetheless, the clinical implications are clear. Keratinocyte responses to mechanical stimuli are vital for effective wound healing and skin regeneration. Developing models that integrate these behaviours across migration, proliferation, and differentiation will be key to designing biomaterials and therapies that align with the regenerative demands of the epidermis.

### 3.2. Electric Fields (EFs)

EFs, particularly endogenous direct current (DC) EFs that arise following skin injury, are potent guidance cues in keratinocyte migration and wound repair. Generated by ionic imbalances across wounded epithelia, these fields typically range from 10 to 60 mV and are oriented such that the wound centre becomes more negatively charged, directing keratinocytes cathodally [91]. This phenomenon, known as galvanotaxis or electrotaxis, has been observed in both individual and collective migration, often superseding guidance signals from chemical gradients due to its stronger directional influence [92]. However, translating this guidance into predictive computational models remains challenging due to the complexity and context-dependence of the electrotactic response.

Experimental studies have shown that EFs accelerate keratinocyte migration via several signalling pathways. For example, EF stimulation downregulates CD9 (a cell-surface protein involved in cell adhesion and migration) via the adenosine monophosphate-activated protein kinase (AMPK) pathway [93]. The objective of the AMPK pathway is to regulate metabolism and cell growth in response to cellular energy status. Other pathways such as EGFR/p38 MAPK/Akt are critical for directed motility [94].

Human keratinocytes exhibit galvanotaxis, migrating directionally toward the cathode in a DC electric field. Gruler et al. proposed a ‘proportional controller’ model to mathematically model the complex, stochastic behaviour of cell migration. It describes a feedback system where the corrective action is directly proportional to the error. This error refers to the deviation from the target direction with the corrective action being a directional change in the cell. Based on time-lapse video recordings, they found that the cell’s migration speed, V and migration angle φ are statistically independent variables [95]. In this model, V is determined by a ‘steerer’ (a controller without feedback), while φ is modulated by an ‘automatic controller’ (a feedback-based system).

The behaviour of this automatic controller is mathematically described following stochastic differential (Langevin) Equation (4):

(4) $$\frac{d\varphi }{dt}=-k_{p}g\left(\varphi ,E\right)+\Gamma (t)$$

where $k_{p}$ is a constant coefficient representing the cellular reaction unit. The g(φ,E) term describes the detection unit’s characteristics, which are a function of the angle **ϕ** and the applied extracellular guiding field, E. This angular dependence is specifically found to be sin(φ), as proposed in the model. $\Gamma \left(t\right)$ represents a stochastic cellular signal, accounting for random movements, and is approximated as ‘white noise’.

The average speed, <V> is crucial for calculating the overall average drift velocity, $<V_{II}>$. This drift velocity is defined as the product of the average speed and the average cosine of the angle, as expressed in Equation (5):

(5) $$<V_{II}>\;=\;<V>\;<cos\varphi >$$

More recent models link sensor (protein or other molecules) redistribution along the cell membrane to galvanotactic migration [96]). Assuming a circular cell, sensor movement is described by Equation (6).

(6) $$\frac{\partial p(\theta ,t)}{\partial t}=\frac{\partial }{\partial \theta }[\kappa \sin\theta p(\theta ,t)]+\frac{\partial^{2}p(\theta ,t)}{\partial \theta^{2}}$$

where p(θ,t) is the probability density of the sensor at membrane angle θ and $\kappa \left(t\right)$ is the rescaled electric field strength (like a Peclet number). It is represented by Equation (7).

(7) $$\kappa \left(t\right)=\frac{\mu E\left(t\right)R}{D}$$

where μ is the sensor’s motility, $E\left(t\right)$ is the field strength, R is the cell radius, and D is the diffusion coefficient.

The sensor diffusion timescale $\tau_{forget}\;=\;\frac{R^{2}}{D}$, quantifies how quickly sensor polarity dissipates, reflecting the time required for sensors to diffuse across the cell membrane.

**Figure 11:**
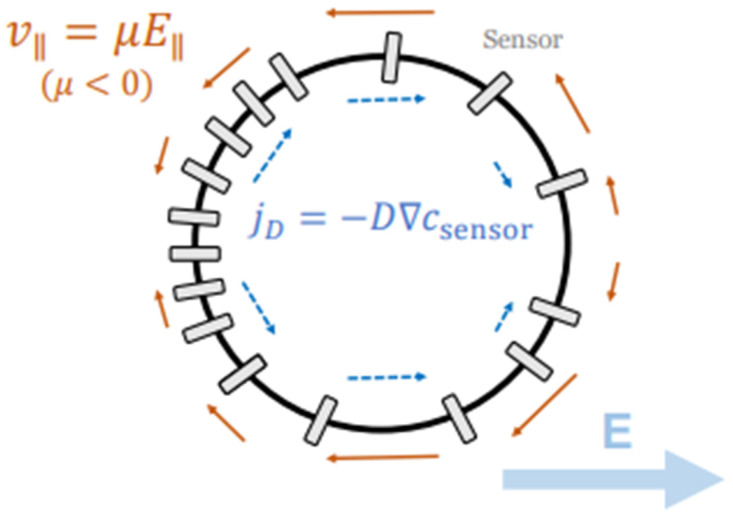
A circular cell is exposed to a uniform electric field (E, bold light blue arrow pointing right), with the anode on the left and cathode on the right. Membrane-bound sensor proteins, initially uniformly distributed, respond to the field via two mechanisms: electrophoresis and electroosmotic flow, both driving leftward movement along the membrane. This net drift is represented by bold orange arrows labelled $v_{II}=\;\mu E_{II}$ where $v_{II}$ is the velocity component parallel to the membrane and μ is the mobility. Opposing this drift, diffusion acts to redistribute the sensors evenly, shown by dotted blue arrows labelled $j_{D}=\;-D\nabla c_{sensor}$, where D is the diffusion coefficient and $c_{sensor}$ is the local concentration of sensors. Over time, sensor accumulation on the left side creates a polarity that enables the cell to detect field direction. Adapted from [96].

This process is illustrated in Figure 11, which shows a circular cell placed in an electric field labelled by symbol *E*, along with the bold arrow pointing to the right. This field acts like a guiding force across the cell, with the anode on the left and the cathode on the right. Charged membrane proteins, called “sensors”, start out evenly spread across the cell’s edge. When the electric field is applied, it causes them to move leftward along the membrane due to two effects: electrophoresis (which pulls charged molecules) and electroosmotic flow (which drags them with fluid). These movements are shown by smaller arrows labelled $v_{II}=\;\mu E_{II}$. The subscript notation (II) specifically refer to the components of velocity and the electric field that are parallel to the cell’s membrane. Counteracting this, diffusion tries to spread the sensors evenly again, shown by $j_{D}=\;-D\nabla c_{sensor}$. Over time, more sensors build up on the left side of the cell, creating a polarity. This imbalance helps the cell detect the direction of the field. In keratinocytes, this polarity leads the cell to migrate toward the cathode (right), which is the first step of galvanotaxis.

The equations discussed in this section are a crucial step in providing mechanistic underpinnings for how EF strength translates into directional sensing and motility. However, a key limitation persists. Experimental studies show that while directionality remains high, collective migration speed drops after approx. 1 hour, even under constant field strength [97]. This adaptation is not well predicted by current models, suggesting missing feedback or fatigue mechanisms.

This is further complicated by the function of the epithelial sodium channel (ENaC). ENaC is a protein channel in the cell membrane that allows sodium ions to pass through, playing a role in fluid balance and electrical signalling. Although ENaC stabilises the protrusions of the cell membrane that extend towards the cathode, its depletion increases speed while impairing directionality [98]. Most models struggle to balance such trade-offs between speed and alignment.

At the collective level, uni-directional EFs accelerate wound closure by increasing coordinated migration, even in diabetic-like keratinocytes [99]. However, PIEZO1, (a mechanosensitive protein) exerts a counter-regulatory effect. It suppresses leader-cell formation and induces cellular retraction, thereby slowing re-epithelialisation [100]. PIEZO1 serves as a braking mechanism for keratinocyte migration in the wound healing process by promoting cellular retraction and inhibiting the formation of leader cells. These are cells at the front that guide collective movement. When PIEZO1 is disrupted, this braking effect is diminished, resulting in faster healing and an increase in leader cell numbers. On the other hand, mutations in PIEZO1 can enhance its activity, intensify the braking effect and ultimately delay wound closure [101]. This demonstrates the essential role of PIEZO1 in the mechanotransduction pathways that influence keratinocyte migration and tissue repair. ABM attempts like [27,102] and ODE models such as [103] attempting to replicate this have captured the impact of retraction strength on migration speed but failed to reflect changes in wound edge geometry suggesting PIEZO1’s influence extends to coordination or intercellular tension, not just local force generation. Nevertheless, this has critical implications for therapeutic development, suggesting that precise modulation, potentially involving inhibition of specific mechanosensors like PIEZO1, could be necessary to accelerate wound healing.

Leader-cell behaviour under EFs highlights further modelling challenges. While Zhang et al. [66] simulated EF-driven directionality propagating across the monolayer, others like Nwogbaga et al. [85] vary in predicting whether leaders remain stable or turn over dynamically based on EF strength. Empirically, EFs trigger biochemical wavefronts that promote the formation of protrusions and leader-cell emergence [104]. However, these spatiotemporal features are hard to capture without models that include local feedback between protrusion mechanics, substrate traction, and cell–cell coordination.

A striking divergence between model and experiment emerges with phosphoinositide 3-kinase (PI3K) inhibition. This refers to blocking the activity of the PI3K enzyme, a key player in many cellular functions including cell growth, proliferation, differentiation, and migration. Single fish keratocytes have been observed to reverse direction under EF when PI3K is blocked, yet large collectives (macro scale) continue cathodal migration [105]. Their proposed “tug-of-war” model explains this by differentiating inner (cathodally biassed) and edge (anodal) cells. Such emergent behaviours reveal the need for hybrid, multiscale models that resolve spatial heterogeneity.

### 3.3. Extracellular Matrix (ECM) Effects

The ECM provides both structural support and a rich source of mechanical and biochemical cues that shape keratinocyte behaviours during migration, proliferation, and differentiation. Keratinocytes attach to components of the ECM using proteins on their surface, such as integrins [106]. These attachments trigger internal signalling processes, known as mechanotransduction. This includes the formation of FAs (structures that connect the ECM to the cytoskeleton), the generation of tension within the cell, and activation of signalling proteins like focal adhesion kinase (FAK) and Rho-GTPases, which help control movement and shape [107]. In addition to bulk stiffness, ECM features like ligand density (such as the spacing of fibronectin), fibre alignment (orientation of ECM fibres like collagen), and porosity critically influence how cells adhere and respond. For example, fibronectin and laminin promote migration and attachment, while collagen I and IV can also impact proliferation and differentiation [108].

**Figure 12:**
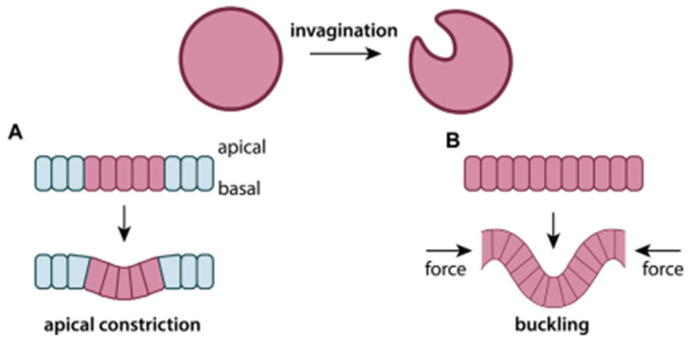
Mechanisms of epithelial morphogenesis. (**A**) Apical constriction: cells contract at their upper edge, inducing curvature and invagination. (**B**) Buckling: external compressive forces deform the tissue layer. Adapted from [109].

Numerous computational models have attempted to simulate how these ECM properties influence keratinocyte behaviour. ABMS and cellular Potts models include ECM parameters like stiffness, fibre (e.g., collagen) elasticity, and pore size to predict migration efficiency [109]. These interactions between cells and the ECM often result in large-scale tissue shape changes, a process known as morphogenesis. Figure 12 illustrates two key mechanisms. Figure 12A is apical constriction, where cells actively contract at their upper edge to drive invagination (folding inward on itself). Figure 12B illustrates buckling, where external compressive forces deform a tissue sheet. These simple schematics provide intuitive insight into how physical forces, whether generated internally or applied externally, can reshape epithelial layers. This mechanical reshaping is often influenced by the ECM, particularly when tissue layers with differing stiffness interact. Compared to neighbouring tissues, if the ECM is too soft, it may deform with the epithelium rather than supporting it. If it is too stiff, it may prevent the epithelium from bending (mechanical mismatch).

These models suggest that deformable cells migrate more easily through soft ECM with small pores, while stiffer ECM supports migration when fibres are well-aligned. They also show that ECM alignment promotes faster and more persistent migration, with leader cells reshaping fibres to guide followers. In hybrid multiscale models, cell behaviour changes with ECM organisation. Specifically, the fibres must be highly aligned for efficient migration [110].

Experimental studies support these modelling insights. In particular, ligand spacing affects collective migration. HaCaT keratinocytes migrate fastest on fibronectin substrates with 50 nm spacing between integrin α5β1 ligands, with both closer (35 nm) and wider (70 nm) spacings reducing speed and coordination [111]. ECM topography, such as fibre alignment, has also been shown to direct cell orientation and movement [112]. In collective migration, ECM composition and ligand density contribute to leader-cell emergence. For example, gradients in ECM stiffness or fibronectin concentration can promote leader-cell formation, while PIEZO1 and angiomotin activity modulate this behaviour by influencing cell tension and signalling [113,114].

However, discrepancies persist. While many models assume a proportional relationship between adhesion ligand density and migration speed, experiments show a non-uniform (stochastic) response with an optimal spacing range. Furthermore, models often assume static ECM environments, whereas in reality, the ECM undergoes dynamic remodelling during wound healing. For example, Fibronectin, Hyaluronic acid, and type III collagen form a temporary matrix that supports keratinocyte movement but is later replaced with a more stable architecture [115]. These changes affect both cell behaviour and matrix mechanics, making accurate predictions more difficult. Additionally, most experiments rely on 2D systems and immortalised cell lines, such as HaCaT, which may not fully represent in vivo responses in complex 3D tissues, as highlighted by [116].

To capture these dynamic influences, we need methods that can convert biochemical signals into clear stress and strain fields. However, this is an objective that continuum and phase-field models alone cannot achieve with adequate spatial detail. Many experimental studies provide correlations between factors, but determining causal mechanisms is challenging without controlled perturbation strategies. Material properties derived from models can directly link physical forces to cellular outcomes Therefore, the next section will focus on FEA, which is the primary solid mechanics framework used. This approach will also integrate these mappings with the chemical and stochastic descriptions developed earlier.

## 4. Finite Element (FE) Modelling of Keratinocytes

FEA has become a foundational tool for simulating keratinocyte mechanics at the single-cell level, offering mechanistic insight into deformation, force transmission, and structural failure under loading. By solving the governing partial differential equations across discretised geometries, FEA enables the estimation of key mechanical parameters. These include Young’s modulus, stress concentration zones, and viscoelastic relaxation time constants, which are experimentally challenging to measure in real time.

Keratinocyte-specific FEA has transitioned from simplified elastic approximations to more biologically informed frameworks capable of capturing the cell’s internal architecture, interfacial loading, and time-dependent behaviours. These models are increasingly integrated into workflows involving real-world measurements. As applications expand to include wound healing, diagnostics, and drug testing, the role of FEA is shifting from theoretical exploration to a practical bridge between in vitro experimentation and predictive simulation.

This section synthesises the current keratinocyte (or related cellular) FEA literature focused at the single-cell resolution. Studies encompass elastic modulus inversion through experimental force and deformation data, internal stress mapping at adhesion sites, and time-dependent modelling of keratinocyte spreading and viscoelastic recoil. By comparing assumptions, outputs, and validation methods across these models, we evaluate where FE predictions align with biological benchmarks and where numerical artefacts or simplifications may skew outcomes.

FEA models are viewed as evolving frameworks rather than definitive predictors. They are influenced by factors such as mesh design, solver strategy, and biophysical assumptions. Each choice impacts how accurately these simulations reflect true cellular mechanics.

### 4.1. Instantaneous Elastic Modulus

FE modelling is widely used to estimate the instantaneous elastic modulus of keratinocytes and related epithelial cells. By simulating deformation under known loads and fitting the outputs to experimental force–displacement data, researchers can determine mechanical properties at both single-cell and tissue levels. This technique spans models of individual cells [117,118], multicellular sheets [119,120], and larger tissue structures like the human hair follicle [121]. Depending on the complexity, these models use tetrahedral or wedge elements to represent cell compartments such as cytoplasm, nucleus, and FA zones [118,120].

**Figure 13:**
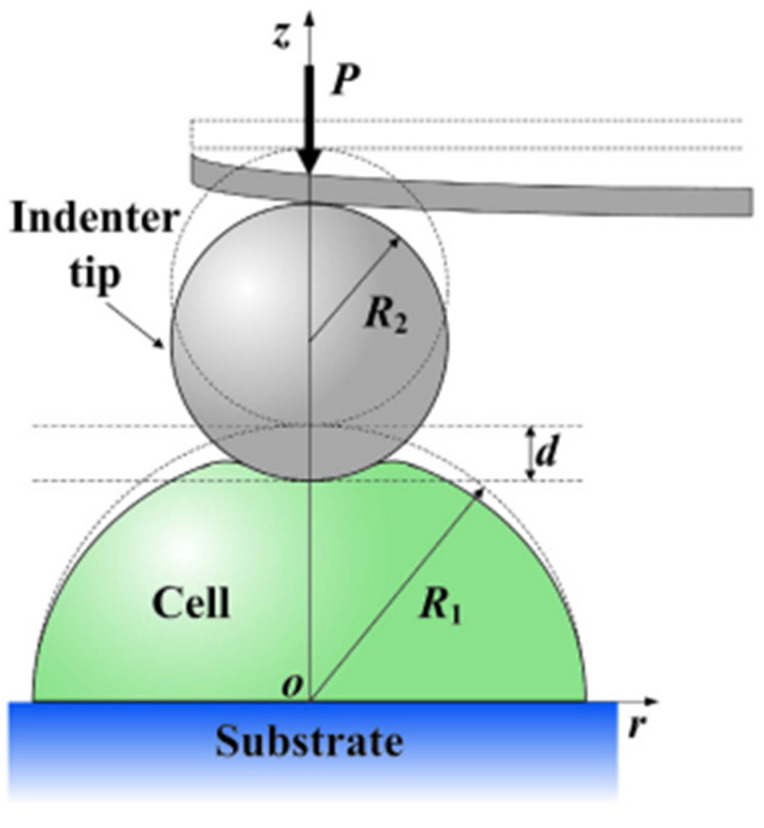
Schematic of AFM indentation on a hemispherical cell. The spherical indenter (radius $R_{2}$) compresses the cell (radius $R_{1}$) adhered to a rigid substrate. The applied force is denoted by P, and the resulting indentation depth is d. The coordinate system used includes the vertical axis z and radial distance r. Adapted from [123].

The basis for many of these measurements is AFM-based indentation. In this setup, a spherical indenter presses against a hemispherical cell adhered to a flat substrate, as illustrated in Figure 13. The force and indentation depth are measured, and the elastic modulus is typically extracted using analytical solutions such as the Hertz model [122].

**Figure 14:**
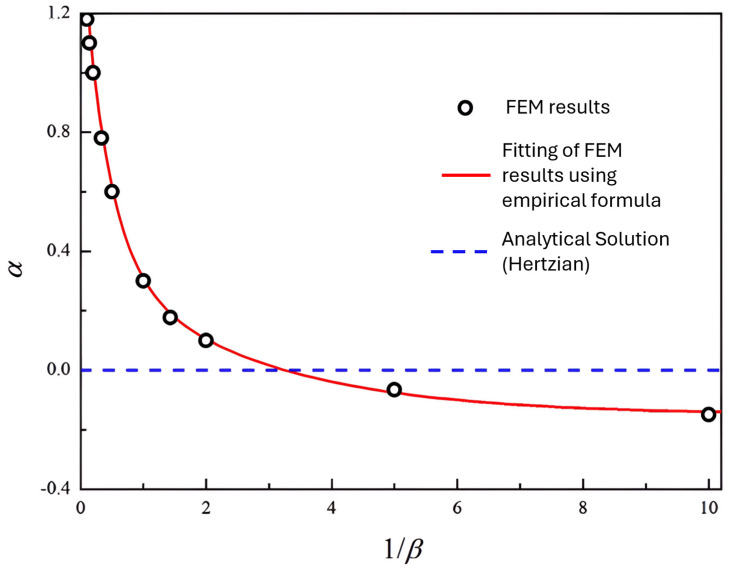
Correction factor α as a function of the radius ratio $\beta =\frac{R_{2}}{R1}$. Deviations from the FEM solution show the limitations of the Hertz model. Adapted from [123].

However, such models assume an idealised contact geometry and can misrepresent the true mechanical response of the cell, especially when subjected to large deformations. Figure 14 demonstrates how another factor, indenter size can also lead to inaccuracies.

The study used FEA to calculate the load, $P_{nH}$, as a function of indentation depth, d for various indenter-to-cell size ratios *β* $=\frac{R_{2}}{R1}$, where $R_{2}$ is the indenter radius and $R_{1}$ is the cell radius (ranging from 0.1 to 10). Using the FEA results, the correction parameter α is determined, which quantifies the difference from the Hertzian equation [124]. This equation assumes small strains and linear elasticity. The FEA results have been used to fit the corrected empirical solution (shown by the red line). The blue dotted line at α = 0 serves as a reference line, corresponding to the analytical Hertzian solution (i.e., no correction for large deformation or surface effects). The graph shows that α depends on the geometric ratio β. The optimal ratio occurs when the two lines intersect (β=0.33 or $\frac{1}{\beta }\;$≈ 3.3).

Deviations from this optimal ratio led to an overestimation (positive α) or underestimation (negative α) of the elastic modulus. When β is much larger, Hertzian models significantly overestimate stiffness. Conversely, for small indenters (β<0.33), stiffness is slightly underestimated. This implies that the choice of indenter radius directly affects the accuracy of modulus estimation, and using an indenter near the critical ratio can help minimise error and excessive distortion. These findings reinforce the need for FEA, which more reliably accounts for complex geometry and deformation, especially outside the narrow conditions where Hertzian assumptions hold.

Material properties are incorporated into FE models through constitutive laws. Linear elastic formulations remain standard for small deformations [118,125], but are insufficient for processes involving larger strain. In such cases, hyperelastic laws like Neo-Hookean or Mooney–Rivlin are preferred due to their capacity to model nonlinear tissue behaviour [126,127]. For hydrated biological materials, porous Neo-Hookean models with added viscosity terms can better capture mechanical complexity [128]. The fitting of these models to empirical data forms the core of inverse FE analysis.

Reported modulus values span a wide range, depending on the biological state, scale, and modelling assumptions summarised in Table 3.

Validation is an essential step in ensuring model accuracy. AFM-based nanoindentation provides force–displacement curves commonly used for model fitting [123,132]. Other validation techniques include inverse FE applied to hydrogel-confined cell deformation [130] and bead displacement in magnetic twisting cytometry [125]. Typically, force or strain predictions from simulations are benchmarked against experimental profiles to assess reliability.

Several factors contribute to variation in modulus values. Keratinocyte stiffness increases substantially with differentiation, changing by over two orders of magnitude in hair follicle studies [121]. Modelling the local effects is also important. Subcellular models may reflect localised stiffness, whereas full-cell models offer averaged estimates [117]. Constitutive choice influences strain response. Neo-Hookean laws underperform beyond 20% strain unless corrected [123]. Furthermore, experimental context matters: soft substrates can cause underestimation unless corrections are applied [133].

Ultimately, these models serve more than a diagnostic function. Changes in stiffness may act as early biomarkers of pathological change [134]. Understanding modulus transitions during keratinocyte maturation can inform scaffold design in skin tissue engineering [135]. FE modelling has also shed light on blistering disorders, where junctional failure affects mechanical integrity [132].

Inverse FE analysis (a method that estimates material properties by fitting simulation results to experimental data) offers a reliable method for measuring the elasticity of keratinocytes. However, understanding a cell’s stiffness alone does not provide insight into where mechanical stresses are concentrated or how those stresses change over time, especially in subcellular structures.

### 4.2. Internal Stress Distribution and Focal Adhesion (FA) Loading

Merely knowing a cell’s modulus does not explain how forces are localised at specific intracellular structures or how they contribute to cellular functions like migration, adhesion, or mechanotransduction. To address this, FE models have been developed to investigate internal stress profiles and FA loading in keratinocytes and related epithelial systems.

These FE models simulate either whole-cell behaviour or subcellular domains. They account for the complex interplay between the cytoskeleton, cell membrane, and the substrate. In whole-cell models, stress is propagated through the cytoplasm and actin networks to adhesion sites at the cell–substrate interface [136]. Subcellular focal contacts have also been modelled explicitly as linear elastic attachments or triangular meshes representing FA regions [137]. In collective migration scenarios, cells are treated as interconnected units within epithelial monolayers, with traction forces mediated through cohesive zone models at the boundaries [138].

**Figure 15:**
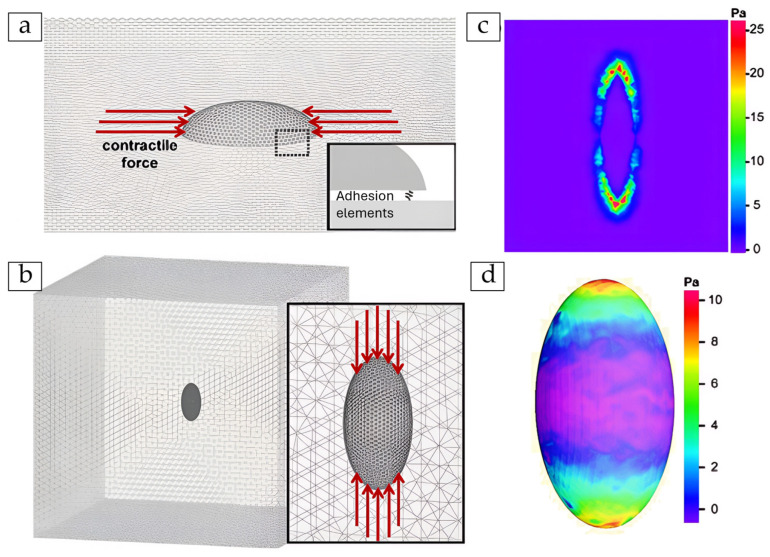
Strain-stiffening cell model implemented in FEA. (**a**,**b**) Geometry and boundary conditions with contractile forces (red arrows) and adhesion elements (zoomed in box). (**c**,**d**) Resulting stress distributions showing how stiffness increases with strain. Adapted from [140].

Importantly, many simulations now account for strain-dependent cell mechanics. Figure 15a–d shows how a strain-stiffening material model is defined within FE simulations, using parameters like the instantaneous modulus $E_{0}$, slope m, and threshold strain $\varepsilon_{0}$. These define how cell stiffness increases as strain accumulates on the edges. This behaviour is often observed in migrating, self-contractile cells [139]. This allows the FE framework to go beyond linear assumptions, enabling more realistic predictions of how cells deform and transmit force to their surroundings.

Element types vary depending on the model’s geometry and focus. For instance, the keratinocyte–liquid crystal interface was discretised into 10,842 triangular elements of approximately 5.7 µm in size [137], while monolayers involved wedge-shaped elements [120]. Three-dimensional solid meshes are often used when simulating cell mechanics with higher resolution [118].

In terms of material properties, linear static stress analysis is the most common method applied to quiescent cells (i.e., cells in a non-dividing, resting state), utilising Hooke’s law [137]. Some models employ hyperelastic materials to represent cellular compliance and couple this with linear elastic substrates to simulate contact mechanics [141]. For tissue-level simulations, isotropic elasticity is often assumed for cells, while cohesive traction–separation laws model the interactions at intercellular or cell–substrate junctions [120].

FE simulations generate spatial maps of stress magnitude and direction, highlighting FA loading and the propagation of intracellular forces. In keratinocytes, FA compressive stresses and total cell–substrate force outputs are consistent with empirical estimates [137]. Epithelial wound models have demonstrated stress localisation at wound edges, supporting the notion that force asymmetry drives directed collective migration [65].

TFM-coupled models can extend this by linking measured surface displacements to calculated traction stresses. TFM-based boundary conditions and validation enable more physiologically grounded modelling, while cytoskeletal networks, especially actin stress fibres are identified as core components for force transmission to FAs. Stress maps derived from TFM reveal dynamic adaptations in force distribution. These adaptations occur in response to changes in cell shape, junction architecture, substrate stiffness, and migratory behaviour [140].

Validation approaches include comparing model predictions to TFM data, shapes of FAs from immunofluorescence images, and observed patterns during wound closure or cell rearrangements [142]. A consistent finding is that mechanical stress accumulates at FA sites, supporting their role in anchoring cells and transmitting force. Hemidesmosomes (HDs), are specialised cell-to-matrix adhesion structures that fix epithelial cells to the underlying basement membrane, providing strong mechanical foundations. Once thought to be passive, they also help regulate these forces by interacting mechanically with FAs and actin fibres [143].

Differences in reported force values often reflect changes in the stiffness of the substrate or whether the model includes just single adhesion points or larger adhesion clusters [144]. These differences show the need for more refined models that can account for various adhesion types and force pathways.

Overall, FE modelling gives a dynamic view of how forces shift and spread across the cell and can offer a much deeper understanding than stiffness values alone. Key studies have been highlighted in Table 4.

To facilitate clarity throughout this table, various abbreviations and technical terms are utilised. The moduli E_0_, E∞, and E_t_ refer to the Instantaneous, Equilibrium, and Tensile Modulus, respectively, while Ec denotes the Compressive Modulus. MPFL refers to the Medial Patellofemoral Ligament. Viscoelastic behaviour is represented using models such as Quasi-Linear Viscoelastic (QLV) and Standard Linear Solid (SLS). The shear modulus is denoted by g, with g_1_ and g_2_ indicating its fast and slow components. Similarly, τ represents relaxation time, with τ_1_ and τ_2_ representing its fast and slow components. Force measurements are quantified in Nanonewtons (nN) and Piconewtons (pN), while viscosity is expressed in Pa·s (Pascal-seconds).

### 4.3. Viscoelastic Response and Active Contraction Dynamics

FE models have evolved to simulate how keratinocytes deform over time and generate active forces. This section explores how time-dependent behaviours, such as stress relaxation and active contraction, are captured computationally. It includes analysis of lamellipodial force generation. This refers to the forces produced by lamellipodia, which are broad, sheet-like protrusions at the leading edge of migrating cells, driven by actin polymerisation.

FE studies in this domain model either single cells, such as osteoblasts or fibroblasts, or epithelial monolayers, using different meshes and laws to capture time-based mechanics. Actin fibres are typically modelled as elastic trusses, while the cytoplasm is treated as a viscoelastic solid [147].

Tetrahedral solid elements and trusses are common at the single-cell scale, with element counts reaching over 58,000 [118]. In tissue-level models, cells are simplified as polygons or wedges and grouped into clusters to capture collective migration [148]. These modelling strategies rely on tailored constitutive laws, summarised in Table 5.

**Figure 16:**
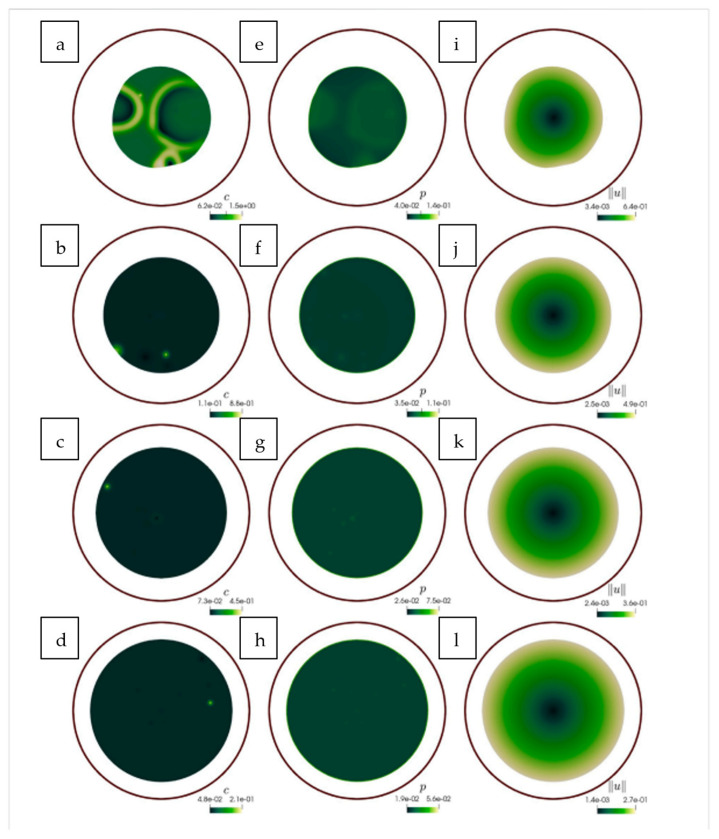
Coupled calcium signalling and viscoelastic response in a contracting domain. (**a**–**d**): calcium distribution has legend c; (**e**–**h**): Herrmann pressure has legend ρ; (**i**–**l**): displacement field has legend u. Moving down rows correspond to increasing mechanochemical coupling strength λ. Adapted from [149,151].

Some models incorporate explicit protrusive forces, mimicking lamellipodia during wound healing [120], or capture anisotropic contraction during morphogenetic events like convergent extension, where a cell converges and extends simultaneously [153]. One approach that directly couples active contraction and viscoelasticity is shown in Figure 16 [149]. The left column (a–d) shows spatial calcium distribution, which triggers contractile stress. The middle (e–h) and right (i–l) columns show the resulting Herrmann (compression) pressure and displacement and captures the viscoelastic deformation of the tissue. Observing from the top row to the bottom, the coupling between calcium and contraction is chosen (represented by a parameter λ) to be stronger. This leads to more pressure and greater deformation. The model shows how a chemical signal like calcium causes physical changes in shape. This helps to understand how stronger feedback leads to greater deformation. This coupling, λ replicates the experimentally observed caused by viscoelastic delays between calcium spikes and cytosolic stretch. It highlights the dynamic interaction between chemical signalling and mechanics.

Key time-dependent properties such as instantaneous and equilibrium moduli, relaxation times, and recoil behaviour, which describe how cells deform under prolonged or cyclic loading, are reported by [154]. Active forces, like those generated by actomyosin networks or protrusive lamellipodia, contribute to coordinated tissue movement and shape change during processes such as wound closure [155,156]. Another critical insight is stress amplification under oscillatory loading, where mechanical forces escalate with frequency [118,157]. This offers a potential explanation for how keratinocytes detect and adapt to dynamic mechanical environments.

Viscoelastic models are typically validated against the following experimental techniques:

1. Stress-relaxation tests, using experimental force–time curves to tune model parameters [158].
2. Laser ablation recoil, where a laser is used to cut part of a cell or tissue, and the subsequent retraction or recoil of the remaining material is measured [159].
3. Time-lapse wound healing, comparing simulated and real closure rates [160].
4. Bioprinted phantoms, which are 3D-printed materials acting as biological substitutes. They test inverse modelling solutions in controlled conditions [161].

A key insight is that viscoelastic behaviour and active force generation are not independent. Active contraction can prestress cells, shaping how they passively respond [32]. While some models isolate passive effects, others integrate active–passive coupling.

A consistent finding across studies is that biological cells and tissues are inherently viscoelastic materials [162,163]. Actomyosin contractility remains the main driver of active forces in keratinocyte models, central to both wound healing and cell remodelling [27,164]. Table 6 summarises representative FE studies that quantify these viscoelastic and active mechanical behaviours.

## 5. Discussion

### 5.1. Summary of Insights and Limitations

Computational modelling of keratinocyte behaviour has matured into a diverse landscape of frameworks, each revealing specific facets of epidermal dynamics. Across these methods, one recurring insight is the convergence of estimated elastic moduli within expected physiological ranges, often between 0.1 and 10 kPa, depending on cell state, substrate, and modelling assumptions [97,106,117]. However, reported time constants for viscoelastic behaviour vary widely, frequently differing by an order of magnitude across studies [49,50,154]. Such divergence stems from differences in constitutive laws, loading conditions, and calibration datasets. Validation remains a major shortfall: while modulus inversion from AFM or micropipette indentation is common, few models incorporate TFM [140] or use stress–relaxation data to benchmark time-dependent responses [158,160].

Several methodological bottlenecks persist. FE outputs often exhibit mesh sensitivity, especially when using linear tetrahedral elements or coarse approximations for cellular geometry [118,137]. Lack of solver transparency in commercial packages limits reproducibility and makes it difficult to trace numerical artefacts. Simplifications in agent-based or continuum models, such as reliance on HaCaT cells, neglect of viscoelasticity, or imposition of static loading. This can further limit physiological realism [90,116]. Many models remain confined to two-dimensional settings, thereby missing out on curvature-dependent phenomena and spatial feedback critical for in vivo scenarios like wound contraction or tumour invasion [115].

A further gap is the limited integration between biochemical signalling, mechanical feedback, and patient-specific heterogeneity. Some models combine R-D equations with cytoskeletal mechanics [39,64]. However, very few of these models incorporate real-time biochemical data, such as protein localisation, or transcriptomic variation (TV) across patient samples [76]. TV refers to the differences in the set of RNA molecules, i.e., transcripts, present in cells, which indicate gene expression patterns that can vary between individuals or different disease states. Multiscale interactions, e.g., how electrotactic polarity at the membrane scales to monolayer flow remain difficult to resolve in current frameworks, especially without mechanochemical coupling or data-driven boundary conditions [66,105].

To address these challenges, several priorities emerge. Hybrid frameworks that combine agent-based decision rules with FE-based mechanical solvers can simulate both intracellular decision-making and extracellular force transmission [61]. Probabilistic modelling of keratinocyte heterogeneity via stochastic rules or population-level Monte Carlo approaches can offer a pathway to better reflect variability seen in single-cell RNA-seq data [77]. There is also a pressing need for standardised benchmarking practices in mechanical modelling, including common indentation protocols, reference phantoms for modulus fitting, and annotated 3D skin constructs for validation under shear and compression [26,138].

Crucially, these improvements will serve as the foundation for integrating advanced computational tools in keratinocyte research. The next section explores how artificial intelligence (AI) and ML are beginning to augment and automate these modelling workflows, helping overcome longstanding bottlenecks in parameter tuning, feature recognition, and multiscale prediction.

### 5.2. Machine Learning (ML) and Artificial Intelligence (AI)

ML and AI are increasingly being integrated into keratinocyte modelling workflows, addressing limitations in data interpretation, parameter calibration, and image-based reconstruction. These tools offer powerful means of extracting quantitative features from complex biological datasets, particularly in high-resolution microscopy and traction force imaging, thereby enhancing the fidelity of FEA and multiscale simulations.

One of the most impactful uses of AI has been in image enhancement and filament network analysis. Techniques like Content-Aware Image Restoration (CARE) have enabled near real-time visualisation of keratin filaments (KFs) at single-fibre resolution, significantly improving the temporal and spatial fidelity of confocal microscopy [169]. This advancement allows researchers to observe keratinocyte cytoskeletal responses to ECM composition and external forces with far greater accuracy than was previously possible. Alongside this, ML segmentation algorithms have been used to quantify cytoskeletal features such as filament orientation, bundling, and density [170]. Deep learning methods applied to actin networks have enabled the high-throughput extraction of filament shape dynamics and deformation trajectories. This offers transferable frameworks for understanding how mechanical cues restructure keratin architecture under both physiological and pathological conditions [171].

**Figure 17:**
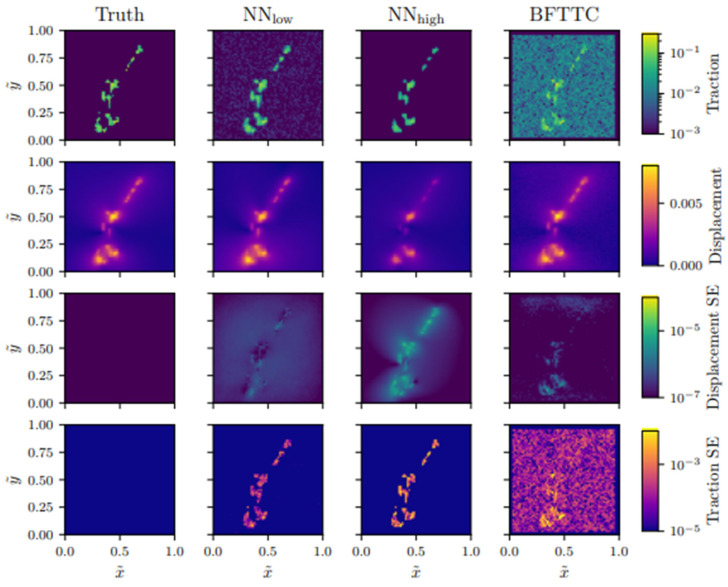
Traction and displacement maps from known data (truth), low-/high-resolution neural networks (NN_low_, NN_high_), and Bayesian FTTC. These maps are arranged in a 4 × 4 grid, with columns representing prediction methods (Truth, NN_low_, NN_high_, Bayesian FTTC) and rows showing (traction fields, displacement fields, squared error, traction squared error). The axes show normalised spatial coordinates and are labelled from 0 to 1. Colour scales indicate magnitudes shown in adjacent labels. NN_low_, NN_high_ can better reconstruct traction patterns with reduced noise outside the cell compared to Bayesian FTTC, though displacement errors are slightly higher. Adapted from [173].

Beyond imaging, ML tools are increasingly used to enhance simulation workflows. Supervised learning models can predict keratinocyte responses to substrate stiffness, ECM ligand spacing, or electric field orientation based on labelled experimental data [172]. When integrated with TFM, ML models can determine subcellular stress patterns. These patterns help reveal mechanical feedback mechanisms, enhancing the biomechanical realism of simulation models [173]. As illustrated in Figure 17 [164], ML-based nural networks (NN_low_, NN_high_) outperform classical TFM solvers by reducing traction noise and better recovering ground truth traction patterns. The state-of-the-art Bayesian FTTC is used for a control reference. These results highlight the potential of AI to resolve inverse problems in biomechanics.

**Figure 18:**
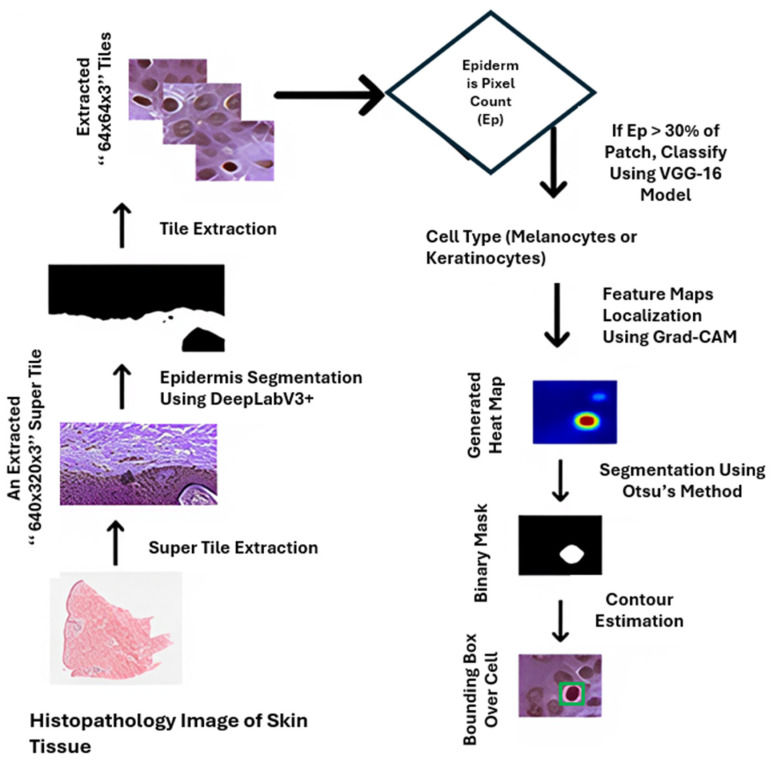
Weakly supervised ML pipeline for cell classification in skin histopathology. The system segments the epidermis, extracts tiles, and classifies cells as keratinocytes or melanocytes. Adapted from [176].

In dermatology, convolutional neural networks (CNNs) have demonstrated the ability to distinguish keratinocytes from melanocytes in histopathology images, improving diagnostic accuracy in conditions such as squamous cell carcinoma and melanoma [174,175]. As shown in Figure 18, a weakly supervised deep learning framework can segment the epidermis, classify cells, and estimate contours from whole-slide images. This pipeline allows automated classification and localisation of keratinocytes and melanocytes. It serves as a foundation for integrating AI into pathology and diagnosis.

These approaches also hold promise for tracking disease progression and evaluating treatment responses. These are capabilities that could be retrofitted into keratinocyte-based digital twin models for predictive simulations [177,178].

**Figure 19:**
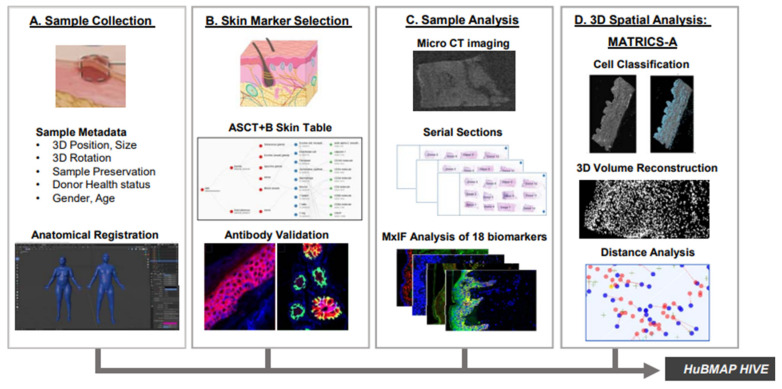
End-to-end AI pipeline for constructing a 3D skin map, from sample collection to spatial analysis and digital twin reconstruction. Adapted from [177].

More broadly, AI can support complex multiscale reconstruction workflows. Figure 19 shows an end-to-end ML pipeline for creating a digital twin of human skin [177]. It combines various aspects like imaging, antibody validation, 3D volume reconstruction, NN_high,_ and spatial analysis. Such platforms offer new opportunities for integrating keratinocyte behaviour into patient-specific predictive models for regenerative medicine and skin disease.

## 6. Future Work and Concluding Remarks

Keratin networks are highly dynamic and context-specific, and current ML models struggle to integrate temporal reorganisation and 3D ECM interactions accurately [179]. As highlighted by recent mapping studies, the structure–function coupling in keratin filament assemblies still resists complete quantification, particularly under cyclic loading or during collective migration [180].

Future advances will depend on coupling high-resolution experimental data with sophisticated computational approaches. FEA stands out as a powerful method for dissecting subcellular stress distributions and viscoelastic properties, but it requires further refinement in meshing strategies, constitutive models, and data-driven parameter estimation. Incorporating ML offers an opportunity not merely for speed gains but for translating complex imaging data directly into mechanical model inputs and for discovering hidden patterns across diverse datasets. AI and ML have emerged not just as analytical tools, but as essential components in the evolution of keratinocyte modelling. Their continued development will be critical in building multiscale, personalised platforms. They are crucial for setting the stage for more robust, predictive, and clinically relevant applications.

This review presents the first modelling-focused synthesis centred on keratinocytes, uniting mathematical formalisms, multiscale simulations, and quantitative mechanical insights under a unified lens. While prior work has explored skin biology broadly, our focus on keratinocyte-specific dynamics reveals clear modelling priorities and challenges that are often distinct from fibroblasts or generic epithelial systems.

R-D and stochastic models have successfully reproduced early-stage signalling patterns and directional migration but often sacrifice biophysical accuracy. Continuum and viscoelastic models have shown how keratinocyte collectives respond to substrate stiffness and EFs but frequently neglect cell-to-cell variability. Our analysis of FE studies shows that mechanical parameters such as Young’s modulus, focal stress, and relaxation time constants vary by over two orders of magnitude depending on mesh resolution, material assumptions, and cytoskeletal representation. This highlights the need for tighter integration between experimental calibration and computational practice.

Critically, we highlight that most models are built using immortalised HaCaT lines, with limited validation against primary normal human epidermal keratinocytes (NHEKs) or patient-derived data. This gap restricts the clinical relevance of many findings. Additionally, only a few studies have been found that account for 3D geometries, layered tissue mechanics, or the evolving composition of the ECM, which are central to skin physiology.

In the future, the combination of GPU-accelerated solvers and ML holds immense promise. AI-driven calibration routines, image-to-mesh translation tools, and surrogate FEA solvers are already beginning to streamline modelling workflows. However, the field must prioritise reproducibility, transparency in code and data, and meaningful cross-validation across various modelling scales.

Keratinocyte mechanics cannot be captured through any single framework. Instead, real progress demands tools that couple discrete migration rules with continuum mechanics and real-world patient data. Only then can modelling evolve from academic insight to clinical utility. We believe this approach has immense potential from powering predictive wound healing to disease diagnosis and digital skin twins for precision dermatology.

## Figures and Tables

**Table 1:** Overview of core stochastic modelling frameworks.

Formalism	Strength	Limitation
ODEs	Computationally efficient, suitable for large systems	Fails to capture stochastic effects, unsuitable for small systems with noise
KMC Methods	Accurate, captures rare events and transitions	Computationally intensive, unsuitable for large systems
Langevin Noise	Computationally efficient, captures intrinsic and extrinsic noise	Less accurate than KMC methods, assumes continuous concentrations

**Table 2:** Overview of advanced stochastic modelling frameworks.

Framework	Linked Core Formalism(s)	Key Uses	Strengths	Limitations
Cellular Potts Model (CPM) [48]	KMC/ODE	Cell shape, adhesion, migration	Captures cell behaviour, polarity, ECM effects	Computationally heavy; overfitting risk
Stochastic Petri Nets (SPNs) [46,52]	KMC/ODE/Langevin	Signalling pathways, fuzzy timing	Structured, handles uncertainty, scalable	Approximate moments only; scaling challenges
Multiscale Stochastic Dynamics (MuTrans) [53]	Langevin/KMC	Cell fate transitions from transcriptomics	Visualises stability landscape; multiscale resolution	Needs large datasets; assumes steady state
Hybrid Methods [54,55]	ODE + KMC/Chemical Langevin Equation (CLE)	Spatial RD systems with variable copy numbers	Efficient multiscale handling; dynamic switching	Complex interfaces; R-D master equation has issues in 2D+
Piecewise Deterministic Markov Processes (PDMPs) [56,57]	ODE + KMC (jumps)	Gene networks, rare-event dynamics	Combines flow and stochasticity; tractable dynamics	Limited analytical solutions; poor fit for continuous noise

**Table 3:** Summary of cell elasticity modulus studies using FEA.

Type of Cell	Experimental Method Used	Estimated Elastic Modulus	Insights
Hair follicle keratinocytes [121]	AFM indentation	30 ± 23 kPa → 11 ± 10 MPa	Large stiffening during differentiation
Epithelial monolayers [120]	Wound closure modelling	approx. 0.2 kPa	Soft phenotype; stress concentration at wound edge
HaCaT (immortalised Human Keratinocytes [129]	AFM nanoindentation with surfactants/peptides	50% decrease with Sodium Lauryl Sulphate; However, the modulus was restored/increased with hydrolysed collagen included	Softening with Sodium Lauryl Sulphate, stiffening with hydrolysed collagen; cytoskeletal protection implied
Cytoplasm of osteoblasts [118]	Cell geometry obtained from confocal microscopy	6.5 kPa	Maxwell model; frequency dependent behaviour
Chondrocytes in hydrogels [130]	Inverse FE fitting to 3D hydrogel deformation	approx. 5 → −20 kPa	Modulus increases with gel stiffness; actin-dependent
Madin–Darby Canine Kidney epithelial domes [131]	AFM force–indentation microscopy	Significantly increased	Stiffer than surrounding monolayers; linked to actomyosin
General cell model [125]	FE model simulating Magnetic twisting cytometry	1 kPa (shear modulus)	Elastic modulus estimation based on bead displacement under applied torque.

**Table 4:** Summary of studies on internal stress using FEA.

Type of Cell	Experimental Method Used	Estimated Stress/Force Output	Insights
Keratinocyte (focal contacts) [137]	Lateral displacement inputs applied along the substrate	14.93 ± 1.9 nN/µm^2^ per FA; Total: 25–90 nN	Stress/displacement decreases quadratically from the force plane
Epithelial cells (monolayer) [120]	FE wound healing simulation	normal > shear stress at localised wound edge.	Captures progressive weakening of a cell–cell junction.
Invasive and non-invasive breast cancer cells. [140]	Traction-force microscopy	Traction stress maps (visualisation of stress)	Cell stiffness and adhesion energy affect force outputs independent of contractility
Keratinocyte + carcinoma [143]	BioID assays, super-resolution imaging	HD’s alter traction and tension	Mechanical coupling exists between HDs and FAs, indicating coordinated force transmission.
FA model [145]	Liquid crystal deformation	Forces distributed around the cells: 25–90 nN	Modulus increases with gel stiffness; actin-dependent
General cell model [141]	TFM-based FE simulation	Intracellular stress distributions; FA loading profiles	Hypoelastic model coupled with linear elastic substrate
Single cell [146]	Fourier Transform Traction Cytometry (FTTC) + particle image velocimetry	Traction stress maps	FA proteins (e.g., talin) essential for cytoskeletal force transmission

**Table 5:** Overview of time-dependent modelling frameworks.

Constitutive Model Type	Purpose/Mechanism	Application
Generalised Maxwell [118]	Captures linear time-dependent stress in viscoelastic cytoplasm.	Osteoblast cytoplasmic response
Kelvin–Voigt [149]	Models long-term elasticity with rapid damping	Embryonic tissue stiffness
QLV + Prony series [127,150]	Describes nonlinear relaxation over short and long timescales	Skin and ligament mechanics
Calcium-dependent contraction [151]	Links Ca^2+^ concentration to active mechanical stress generation and viscoelasticity	Embryonic epithelial contraction
Cohesive zone [152]	Simulates adhesion dynamics at junctions under time-varying forces	Accurately predicts initiation, growth, and rupture of adhesions under cyclic stresses

**Table 6:** Summary of studies of time-dependent cellular behaviour using FEA.

Type of Cell	Experimental Method Used	Estimated Viscoelastic/Active Parameters	Insights
Murine MC3T3-E1 osteoblasts [165]	AFM	Creep equilibrium times: almost instant at the edge, approx. 3–4 s at cytoplasm, approx. 4–5 s at nucleus Instantaneous elastic modulus (E_0_): edge 8 kPa, nucleus 1.7 kPa, cytoplasm 2.5 kPa	Nucleus is softest with slowest creep. Cytoskeletal remodelling likely causes delayed viscoelastic recovery.
Embryonic epithelial tissue [31,149]	Area contraction + Ca^2+^ modulation	Shear viscosity: 3790 Pa s Bulk Viscosity: 550 Pa s	Active contraction tied to intracellular calcium concentration.
Skin tissue (rat) [48,150]	Stress-relaxation test	QLV + Prony Parameters (back skin): $E_{0}$ ≈ 12.7 MPa, g_1_ = 0.3, g_2_ = 0.25 τ = 0.062s	approx. 70% relaxation Highly viscoelastic; stronger relaxation in back skin than abdomen.
Epithelial monolayer [166]	Wound healing experiments	Edge protrusion force ≈ 2 pN; Protrusion force elsewhere ≈ 0–0.3 pN	Promotes faster wound closure; active tug-of-war at boundary.
Epithelial embryos of fruit fly embryos (Drosophila melanogaster) [148]	Laser ablation (microsurgery)	Viscosity ≈ 10 Pa·s; Tension ≈ 1.9 nN; Stress ≈ 100 Pa	Adding viscoelastic rods improves the model’s accuracy in simulating recoil behaviour after wounding.
Bioprinted skin substitute with keratinocytes, fibroblasts etc. [167]	Water-based process to create hydrogel bioink. Variable shear rates with a range of 0.1–1000 s were then applied	$E_{c}$$\text{:}2.6\;\pm \;0.3\;\mathrm{kPa}\;\mathrm{at}\;37\;{}^{\circ}C\;E_{t}$ increased from 7.2 ± 0.6 kPa to 14.7 ± 1.3 kPa after 24 h at 37 °C	In vivo, the bi-layer constructs achieved significant wound healing and tissue integration after 28 days.
Heterogeneous materials (single inclusion phantom) [168]	Ultrasound via excitation through compressional waves to assess tissue characteristics	$E_{0}$ = 10–110 kPa; τ = 0.1–11.5 s. Maxwell SLS model used to fit creep curves from FE simulations.	Measurement accuracy drops when E or τ of inclusion material differs >10% from background. Boundary zones show greatest error.
3D-Printed Tissue-like Materials (J750 Shore) [166]	uniaxial tensile stress–relaxation tests	$\mathrm{Neo}\text{-}\mathrm{Hookean}\;\mathrm{fit}\;\mathrm{with}\;3\;\mathrm{term}\;\mathrm{Prony}\;\mathrm{series}\;(\mathrm{Shore}\;30\;\mathrm{material})\;E_{0}$ = 0.87 ± 0.02 MPa, g_1_ = 3.00, τ_1_ = 0.49 s; g_2_ = 0.23, τ_2_ = 11.0 s; g_3_ = 0.12, τ_3_ = 49.2 ± 4.67 s;	3D-printed materials show Neo-Hookean behaviour, lacking the anisotropy and strain stiffening of real tissues.

**Figure A1:**
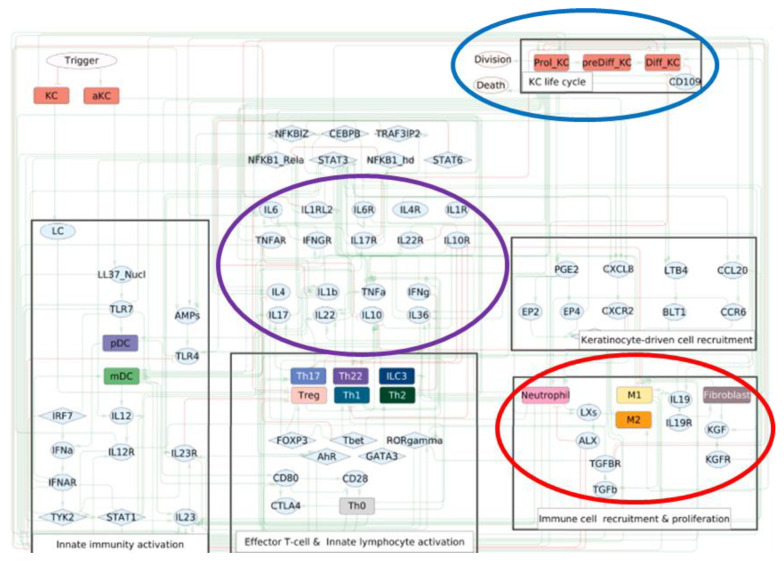
PsoriaSys Boolean model, showing cell types, keratinocyte states, and molecular signals with activating (green links) and inhibitory (red links) driving psoriasis dynamics [60]. cell types shown in a red circle (e.g., neutrophils, pDCs, Th17), keratinocyte states shown in a blue circle (e.g., proliferating keratinocytes [Prol_KC], activated keratinocytes [aKC])), molecular entities shown in a purple circle (e.g., IL-17 is a liganf, TNFa, STAT3).

## Data Availability

The authors confirm that the data supporting the findings of this study are available either within the article, its references.

## Abbreviations

FBP1	Fructose-1,6-Bisphosphatase 1
REG3A	Regenerating Islet-Derived Protein 3 Alpha
ML	Machine Learning
IF	Intermediate Filaments
LAD	Lamina-Associated Domains
ABM	Agent-Based Models
SCC	Squamous Cell Carcinoma
FEA	Finite Element Analysis
LED	Light Emitting Diode
micro-PIV	micro-Particle Image Velocimetry
ATP	Adenosine Triphosphate
EGF	Epidermal Growth Factor
EGFR	Epidermal Growth Factor Receptor
PDE	Partial Differential Equations
CPM	Cellular Potts Model
PDMP	Piecewise Deterministic Markov Processes
DC	Direct Current
PBC	Particle-Based Compass
ERK	Extracellular Signal-Regulated Kinase
LIC	Line Integral Convolution
PA	Polyacrylamide
PDMS	Polydimethylsiloxane
EFs	Electric Fields
AMPK	Adenosine Monophosphate-Activated Protein Kinase
RNA-seq	Single-Cell RNA Sequencing
CNN	Convolutional Neural Networks
AI	Artificial Intelligence
NHEK	Normal Human Epidermal Keratinocytes
GPU	Graphics Processing Unit
SPNs	Stochastic Petri Nets
MuTrans	Multiscale Stochastic Dynamics
MAPK	Mitogen-Activated Protein Kinase
FAK	Focal Adhesion Kinase
PI3K	Phosphoinositide 3-Kinase
KFs	Keratin Filaments
CARE	Content-Aware Image Restoration
TV	Transcriptomic Variation
NN	Neural networks
ODEs	Ordinary Differential Equations
KMC	Kinetic Monte Carlo
CLE	Chemical Langevin Equation
R-D	Reaction–Diffusion
ECM	Extracellular Matrix
Prol_KC	Proliferating Keratinocyte
Th	T-helper
IL-17	Interleukin 17
TNFα	Tumour Necrosis Factor Alpha
HaCaT	Human Adult Low Calcium Temperature
AFM	Atomic Force Microscopy
FE	Finite Element
TFM	Traction Force Microscopy
FTTC	Fourier Transform Traction Cytometry
FA	Focal Adhesion
HD	Hemidesmosome
QLV	Quasi-Linear Viscoelastic
MPFL	Medial Patellofemoral Ligament
SLS	Standard Linear Solid
